# Utility of Host Delivered RNAi of Two FMRF Amide Like Peptides, *flp-14* and *flp-18*, for the Management of Root Knot Nematode, *Meloidogyne incognita*

**DOI:** 10.1371/journal.pone.0080603

**Published:** 2013-11-06

**Authors:** Pradeep Kumar Papolu, Nagavara Prasad Gantasala, Divya Kamaraju, Prakash Banakar, Rohini Sreevathsa, Uma Rao

**Affiliations:** 1 Division of Nematology, Indian Agricultural Research Institute, New Delhi, India; 2 National Research Centre on Plant Biotechnology, Indian Agricultural Research Institute, New Delhi, India; University of Louisville, United States of America

## Abstract

Root knot nematode, *Meloidogyne incognita*, is an obligate sedentary endoparasite that infects a large number of crop species and causes substantial yield losses. Non-chemical based control strategies for these nematodes are gaining importance. In the present study, we have demonstrated the significance of two FMRFamide like peptide genes (*flp-14* and *flp-18*) for infection and development of resistance to *M. incognita* through host-derived RNAi. The study demonstrated both *in vitro* and *in planta* validation of RNAi-induced silencing of the two genes cloned from J2 stage of *M. incognita*. *In vitro* silencing of both the genes interfered with nematode migration towards the host roots and subsequent invasion into the roots. Transgenic tobacco lines were developed with RNAi constructs of *flp-14* and *flp-18* and evaluated against *M. incognita*. The transformed plants did not show any visible phenotypic variations suggesting the absence of any off-target effects. Bioefficacy studies with deliberate challenging of *M. incognita* resulted in 50-80% reduction in infection and multiplication confirming the silencing effect. We have provided evidence for *in vitro* and *in planta* silencing of the genes by expression analysis using qRT-PCR. Thus the identified genes and the strategy can be used as a potential tool for the control of *M. incognita*. This is the first ever report that has revealed the utility of host delivered RNAi of *flps* to control *M. incognita*. The strategy can also be extended to other crops and nematodes.

## Introduction

Plant-parasitic nematodes are responsible for global agricultural losses amounting to an estimated $157 billion annually [1]. Root knot nematodes (*Meloidogyne* species) are the most economically important group of plant parasitic nematodes (PPNs) worldwide, attacking nearly every food and fiber crop grown [2]. The potential host range of these obligate, sedentary endoparasites encompasses more than 3000 plant species [3]. *M. incognita*, representing the most widespread species, is found in every country in which the lowest temperature is more than 3°C and is therefore possibly the most damaging crop pathogen in the world [4]. Chemical nematicides have been discontinued due to their detrimental environmental effects and other alternative management approaches are not sufficient demanding an urgent need to design novel management strategies. Since transgenic technology has offered potential new avenues for crop improvement programs, it could be useful for designing nematode resistant crops.

FMRFamide (Phe-Met-Arg-Phe) is a neuropeptide from a broad family of FMRFamide-related peptides (FaRPs) all sharing an -RFamide sequence at their C-terminus. FMRFamide like peptides (FLPs) belong to FaRPs comprising the largest family of neuropeptides in nematodes. Most of the structural information of FLPs has been generated from *Caenorhabditis elegans* and the functional data of FLPs comes from the nematode physiological model, *Ascaris suum*. The studies on FLPs in *A. suum* indicate that their vital responsibility for the modulation of nerve and muscle activity in a concentration dependent and reversible manner [5,6]. They are responsible for modulating pharyngeal muscle activity in *A. suum* [7]. Similarly, FLPs are also accountable for multiple responses in the ovijector musculature ranging from transient excitation to persistent or transient inhibition [8,9]. Therefore, these peptides and their associated processes are considered as potential control targets for parasitic helminths [10-12]. As in the case of *A. suum*, presence of FMRFamide-like immunoreactivity has also been demonstrated in the nervous system of PPNs, *Globodera pallida* and *G. rostochiensis* [13]. Recently it has been reported that *flp-32* in *G. pallida* was responsible for the modulation of locomotory behavior and putatively interacted with at least one novel G-protein coupled receptor [14].

FLPs are shown to be present in different parasitic nematodes having similar structural homologues and functions. Consequently, disruption of these activities in PPNs represents an attractive novel control strategy as it would interrupt the worm’s ability to hatch, migrate through the soil to reach the host, feed on the host tissue and also to mate. So far, 19 FLPs have been identified in *M. incognita* based on conserved FMRFamide domain analysis of the ESTs and the whole genome sequence by comparative genomics [1], out of which six have transcriptional confirmation (*flp*-1, 7, 12, 14, 16, 18 – NCBI GenBank database). MSA (Multiple Sequence alignment) of these six confirmed *flp* genes showed low nucleotide sequence level conservation among them although they share a common RF-amide sequence at C-terminus. Their uniqueness could therefore be harvested at developing sequence specific knockout module by dsRNA method to avoid off target effects. Further, accurate physiological roles of only few of the FLPs are known in *C. elegans* and *A. suum*. However, major information on these FLPs is lacking in *M. incognita* probably due to their small size and obligate relationship with the host which limits the use of standard physiological techniques. Nevertheless, silencing of *flp-14* and *flp-18* in *M. incognita* has been reported to interrupt the migration of worms in response to the root exudates [15,16]. In view of this, we have selected these two genes with no sequence similarity between them in the present study.

RNA interference (RNAi), the conserved phenomenon of gene silencing mediated by double-stranded RNA (dsRNA), represents a promising molecular tool with potential applications in both the functional genomics and control of PPNs. The ability to specifically knock down a selected mRNA transcript allows the investigation of gene function and interaction, in addition to the validation of putative control of targets through loss of function phenotype analysis. The study of gene function through RNAi is well documented for many PPNs including *G. pallida* and *M. incognita* [3,17-23]. In addition to these reverse-genetics applications, *in planta* RNAi has shown potential as a method of PPN control with several published accounts of such an approach in both cyst and root knot nematodes [14,22,24-28]. The present paper deals with the unequivocal utility of host delivered RNAi of the two *flp* genes, *flp-14* and *flp-18* and their effect on *M. incognita.*

## Materials and Methods

### Nematodes

A pure culture of Indian isolate of *M. incognita* was maintained on a susceptible egg plant variety Pusa purple long under green house conditions. Nematode infected plants were harvested at appropriate intervals to collect feeding females (FF) and egg masses. These egg masses were used for hatching the infective second stage juveniles (J2s); additionally eggs were also collected separately. 

### Cloning and sequencing of *flp-14, flp-18* of *M. incognita*

Total RNA was extracted from J2s using NucleoSpin total RNA Kit (Macherey-Nagel, Germany). Extracted RNA was assessed for quality and quantity using Nanodrop ND-1000 spectrophotometer (Thermo Scientific) and Agilent 2100 Bioanalyzer with RNA 6000 nanokit (Agilent Technologies). RNA with an RNA integrity number (RIN) of 8.0 was used for cDNA synthesis. Further, 300 ng of the RNA sample was reverse transcribed to cDNA using cDNA synthesis Kit (Superscript VILO, Invitrogen). A 284 bp and 407 bp region of *flp-14* and *flp-18* (GenBank: AY907829, AY729022) respectively were amplified separately from the cDNA using the primers as given in table 1. 

**Table 1:** Primers used for PCR amplification of *flp-14* and *flp-18*.

**Name**	**Accession**	**Sequence (5’ --- 3’)**	**Length (bp)**	**Tm (° C)**
***flp-14* F**	AY907829	AACGCAAATACTCGTGCTTTCT	284	60
***flp-14* R**		TATGCAGCCATCTAACAATTCCT		
***flp-18* F**	AY729022	CGATGAAAGACCAAAACGTG	407	60
***flp-18* R**		ACGATGATGGAAAGGAATGG		

The two PCR products were cloned separately into pGEM-T easy cloning vector (Promega) using standard protocol. Freshly prepared competent cells of *Escherichia coli DH5α* were transformed with the recombinant plasmids. Positive clones were selected by blue white colony screening and colony PCR. Plasmids were extracted from recombinant colonies and confirmed by restriction digestion with EcoRI. The positive clones were custom sequenced by ABI SOLiD sequencing system [29]. 

### Differential expression of *flp-14, flp-18* in developmental stages of *M. incognita* using qRT-PCR

RNA was extracted from three different stages (eggs, J2s, females) of *M. incognita* and cDNA was prepared by using 300 ng of RNA. Quantitative Real-time (qRT-PCR) was performed using SYBR Green I technology in realplex^2^ thermal cycler (Eppendorf). A master mix for each of the samples was prepared by mixing SYBR Green I, blue dye, ROX passive reference and stabilizers and PCR Core Reagents (Eurogentec). In this study, *18S rRNA* was used as an internal reference gene; 1.5 ng of cDNA and 750 nM each of the specific primers were added in a final volume of 10 µl (Table 2). The amplification reactions were carried out at a hot start of 95° C for 5 min, followed by 40 cycles of 95° C for 15 s and 60° C for 1 min in a qPCR high profile non skirted white 96-well plate (Eurogentec). Specificity of amplification was assessed by disassociation or melt curve analysis at 60-95° C after 40 cycles. Three biological replicates and three technical replicates were used for each of the samples. The mean ct values were taken for calculating the fold change (2^-ΔΔCT^) [30].

**Table 2:** Real-time PCR primers used for expression analysis.

**Name**	**Sequence (5’ --- 3’)**	**Length (bp)**	**Tm (° C)**
***flp-14* RT F**	GCGAGTCCATGTGTAGCAGCTAAT	117	60
***flp-14* RT R**	GGGAGATGAAGAACGTTTACTACTTTGCC		
***flp-18* RT F**	AGGATGACTTATTGCGCCAGGA	185	60
***flp-18* RT R**	TTCCTTTACCGAATCTGAGCACGC		
***18SrRNA* Tobacco RT F**	CGCGCGCTACACTGATGTATTCAA	172	60
***18SrRNA* Tobacco RT R**	TACAAAGGGCAGGGACGTAGTCAA		
***18SrRNAM.incognita* RT F**	TCAACGTGCTTGTCCTACCCTGAA	155	60
***18SrRNAM.incognita* RT R**	TGTGTACAAAGGGCAGGGACGTAA		

### Preparation of dsRNA

The *flp-14, flp-18* and GFP gene fragments were PCR amplified from respective pGEM-T clones using M13 primers. Gel purified PCR products were used as template for synthesizing sense and antisense strands of all the three genes using T7 and Sp6 transcription kits (Ambion). dsRNA was synthesized by mixing the two ssRNAs and incubating at 65° C for 5-10 min followed by 37° C for 30 min; confirmed on 1% agarose gel and stored at -20° C until further use. dsRNA of an unrelated green fluorescent protein (GFP) of 750 bp cloned in pGEM-T vector was synthesized to be used as a negative control.

### In vitro RNAi of flp-14 and flp-18

Freshly hatched J2s were collected and used for dsRNA uptake studies as described [17]. About 15,000 freshly hatched J2s were soaked in 100 µl of RNAi soaking solution containing 3 µg dsRNA along with 50 mM Octopamine and incubated for 24 h in dark on a slowly rotating vertical platform at room temperature. In control samples, the J2s were incubated in the same solution without dsRNA. In order to demonstrate target specific silencing, worms were also soaked in dsRNA of GFP. After 24 h the nematodes were washed thrice in sterile spring water by gently inverting the tubes for resuspension of the worms followed by a brief centrifugation for collection of worms. The nematodes were examined under the microscope for mobility, normal behavior and used for further experimental studies. The concentration of the worms was adjusted as 20 worms per 10 µl of water. 

We performed an attraction and migration assay on pluronic gel for determining the effect of gene silencing [31]. For this, 1000 J2s were placed in the middle of a 2 inch diameter petriplates; 5 ml of 23% pluronic gel F-127 was added and the plates were rotated gently. This helped in mixing of the worms with the gel and uniform spreading of both the gel and worms all over the plate. Following this, five day old tomato seedling (variety Pusa Ruby) was placed on the gel in a corner and the plates were incubated at room temperature for the gel to solidify. Migration of J2s was monitored at 4 and 8 h. At 24 h, infected roots were stained with acid fuchsin to observe the number of J2s that had penetrated into the roots [32]. At this interval, nematodes around the roots were also counted. Six replicates were taken for each of the treatments. Data were analyzed by one-way ANOVA and CRD test for significance followed by Duncan’s multiple-comparison test with significance level at *P*<0.05 and *P<0.01* using SAS software (version 9.3).

In order to analyze the expression of target genes in the dsRNA-treated nematodes, total RNA was extracted from worms treated with target dsRNA, dsRNA of unrelated control (GFP) and control (worms soaked in water). cDNA synthesis and qRT-PCR analysis was performed as mentioned above. Three biological and three technical replicates were taken for each of the samples. *18S rRNA* was used as a reference gene. Fold change was calculated using 2^-ΔΔCT^, expressed as percentage and student t test was performed.

### Development of RNAi constructs of *flp14* and *flp18* for *in planta* validation

The pK7GWIWG2(I) vector (RNAi GATEWAY ready) [33] was obtained from VIB Department of Plant Systems Biology, Ghent University, Belgium (http://www.psb.ugent .be/ gateway/). Partial sequences of *flp-14*, *flp-18* (284bp, 353bp) were initially amplified from pGEM-T clones and cloned separately into the entry vector (pDONR 221). Primer details are given in table 3. These gene fragments were subsequently cloned into GATEWAY ready pK7GWIWG2 (I) RNAi vector in sense and antisense orientation intervening with an intron by GATEWAY recombination based cloning (Invitrogen) (Figure S1). These RNAi constructs were transformed to *E. coli* (DH5α) cells and colony PCR was performed using three different sets of primers (gene specific forward and reverse; CaMV 35S promoter forward and attB2 reverse; CaMV 35S terminator forward and attB2 reverse; *nptII* forward and reverse primers) to confirm the orientation of the target gene. The PCR products were sequenced and BLAST analysis was done to ensure that PCR reaction specifically amplified the target genes. Further, *Agrobacterium tumefaciens* strain LBA4404 was transformed with the recombinant constructs by electroporation and used for validation studies in *Nicotiana tabacum*. 

**Table 3:** Primers used for cloning of *flp-14* and *flp-18* in RNAi vector and analysis of transgenic tobacco plants.

**Name**	**Sequence (5’ --- 3’)**	**Length (bp)**	**Tm (° C)**
**GV *flp-14* F**	GGGGACAAGTTTGTACAAAAAAGCAGGCTAACGCAAATACTCGTGCTTTCT	342	60
**GV *flp-14* R**	GGGGACCACTTTGTACAAGAAAGCTGGGTATGCAGCCATCTAACAATTCCT		
**GV *flp-18* F**	GGGGACAAGTTTGTACAAAAAAGCAGGCTGACTCTTTGGAAACGTTCACTT	411	60
**GV *flp-18* R**	GGGGACCACTTTGTACAAGAAAGCTGGGTGATGATGGAAAAGGAATGGCTA		
**CaMV35S Promoter**	TCCTTCGCAAGACCCTTC		
**CaMV35S Terminator**	CCTTATCTGGGAACTACTCACAC		
**attB1**	GGGGACAAGTTTGTACAAAAAAGCAGGCT		
**attB2**	GGGGACCACTTTGTACAAGAAAGCTGGGT		
**nptII F**	CAA TCG GCTGCTCTCATGCCG	750	60
**nptII R**	AGGCGATAGAAG GCGATGCGC		

### Plant material and growth conditions

Seeds of tobacco variety, Petite Hawana, were surface sterilized with 70% alcohol for 2 min and washed with sterile distilled water; subsequently, they were treated with 0.1% HgCl_2_for 10 min and washed with sterile distilled water to remove the sterilant. Later the seeds were germinated on half strength MS medium with 1.5% sucrose and 0.7% agar (pH 5.8). The seedlings were maintained in 16 hours light and 8 hours dark.

### In planta RNAi of flp-14, flp-18 of *M. incognita* using tobacco

Leaf explants of 1cm^2^ cut from young tobacco leaves were used for *Agrobacterium* mediated transformation. Explants were kept in pre-cultivation medium for 3 days (MS+ 0.1mg L^-1^ NAA + 2 mg L^-1^ BAP). Subsequently, they were infected with *Agrobacterium* (LBA4404) harboring the respective RNAi constructs for 5 min. Later, the leaf explants were blotted dry on sterile tissue paper and co-cultivated for 2 days on co-cultivation media (MS+ 0.1mg L^-1^ NAA + 2 mg L^-1^ BAP). Subsequently, they were incubated on selection medium (MS+ 0.1 mg L^-1^ NAA + 2 mg L^-1^ BAP + 100 mg L^-1^ kanamycin+ 250 mg L^-1^ cefotaxime). After 30 days, shoots produced from the explants were sub cultured at 10-15 days interval into fresh selection medium. The elongated shoots were further transferred to rooting media (1/2 MS + 100 mg L^-1^ kanamycin+ 250 mg L^-1^ cefotaxime). The resulting plants with well established roots were hardened and transferred to National Phytotron Facility of IARI, New Delhi for further growth and production of T_0_ seeds. 

### Molecular analysis of the primary transgenics

#### DNA extraction and PCR confirmation of T_0_ plants

Genomic DNA was extracted from the fresh leaves of all the primary transgenic events using Nucleospin Plant II DNA extraction kit (Macherey-Nagel). Preliminary evaluation of the *flp-14, flp-18* transformants at the molecular level was carried out by PCR analysis using different sets of primers (gene specific forward and reverse; CaMV 35S promoter forward and attB2 reverse; CaMV 35S terminator forward and attB2 reverse; *nptII* forward and reverse primers) (Table 3). The PCR products were resolved on 1.2% agarose gel.

### Genomic southern analysis

To confirm the T-DNA integration, DNA (15 µg) from the PCR positive plants was digested with *Sac*I that cuts once in the T-DNA. Digested DNA was resolved on 0.8% agarose gel and then transferred onto a nitrocellulose membrane (BioRad Zeta probe). For probing, 284 bp and 353 bp fragments of *flp-14*, *flp-18* genes respectively were used. The DNA fragments were labeled with α[^32^P]-dCTP by mega prime DNA labeling kit (Amersham Pharmacia Biotech) and hybridization was carried out at 65° C for 18 h. The membranes were later washed at 65° C with 3× SSC and 0.1% SDS, followed by 0.5× SSC and 0.1% SDS, for 30 min each. They were finally washed with 0.1× SSC and 0.1% SDS for 30 min [34], exposed to Fujifilm (Kodak) for 36 hr and developed. 

### Molecular analysis of T_1_ plants

T_0_ seeds of plants of both the genes were germinated on MS+100 mg L^-1^ kanamycin and shoots were transferred to ½ MS medium along with 0.25 mg L^-1^ GA3 (Duchefa) and 100 mgL^-1^ kanamycin. Plants with optimum growth of shoots and roots were then transferred to 150 ml pots filled with autoclaved soil and soilrite in the ratio of 3:1. These plants were kept in the growth chamber (Labtech) at 27° C, 70% RH, 16h light and 8h dark for 10-15 days. DNA was extracted from the fresh leaves of these plants as explained above and was analyzed by PCR using different sets of primers as in the case of T_0_ plants (Table 3). PCR confirmed T_1_ plants of different events were further subjected to real-time PCR and bioefficacy analysis. qRT-PCR analysis was carried out using primers specific to the target genes and *18S rRNA* (reference gene) as stated above (*in vitro* RNAi of *flp-14*, *flp-18*). ΔCT values were later calculated by determining the difference between the ct mean of the target gene and the reference gene for obtaining the average ΔCT values. Representative samples of T_1_ plants expressing dsRNA of *flp-18* were also confirmed by northern analysis for the presence of both dsRNA and siRNA. For this, total RNA was extracted from the leaves using NucleoSpin total RNA Kit (Macherey-Nagel) while, that of siRNA was extracted using NucleoSpin small RNA Kit (Macherey-Nagel). Both the total RNA and siRNA were resolved on 30% denaturing PAGE gel and then transferred onto a nitrocellulose membrane (BioRad Zeta probe). For probing, 353 bp fragment of *flp-18* gene was used. The DNA fragment was labeled with α[^32^P]-dCTP by mega prime DNA labeling kit (Amersham Pharmacia Biotech) and hybridization was carried out at 42° C for 18 h. The membranes were later washed at 65° C with 3× SSC and 0.1% SDS, followed by 0.5× SSC and 0.1% SDS, for 30 min each. They were finally washed with 0.1× SSC and 0.1% SDS for 30 min [34], exposed to Fujifilm (Kodak) for 36 hr and developed. 

### Efficacy studies of T_1_ plants expressing dsRNA of *flp-14, flp-18* against *M. incognita*

The roots of 15 days old transgenic plants kept in growth chamber were inoculated with approximately 300 freshly hatched J2s. The nematode inoculated plants were grown at 27° C, 70% relative humidity, 16h light and 8h dark for 30 days to complete the lifecycle. After 30 days, the roots were washed free of soil and total number of galls, females, egg masses and eggs per egg mass were counted for each plant. These were compared with the wild type plants which were also inoculated and grown under similar conditions. Six replicates were used in the study. The observations were made for all the replicates separately and subjected to analysis of variance (one-way ANOVA). Observations were reported as significant or non-significant using the CRD test, followed by Duncan’s multiple-comparison test with significance level at *P*<0.05 and *P< 0.01* using SAS software (version 9.3).

### Expression analysis of the target gene in the nematode extracted from transgenic plants

Mature females were extracted from both the wild type and transgenic plants and then analyzed by qRT- PCR to assess *flp-14* and *flp-18* transcript accumulation. To determine target specific silencing effect, expression of *flp-18* was analyzed in the females collected from transgenic plants expressing dsRNA of *flp-14* and vice versa. *18S rRNA* was used as reference gene. Six biological and three technical replicates for *flp-14* and *flp-18* were taken for the study. Fold change was calculated using 2^-ΔΔCT^, expressed as percentage and student t test was performed.

## Results

### Cloning and differential expression of *flp-14, flp-18* of *M. incognita*

**Figure 1:**
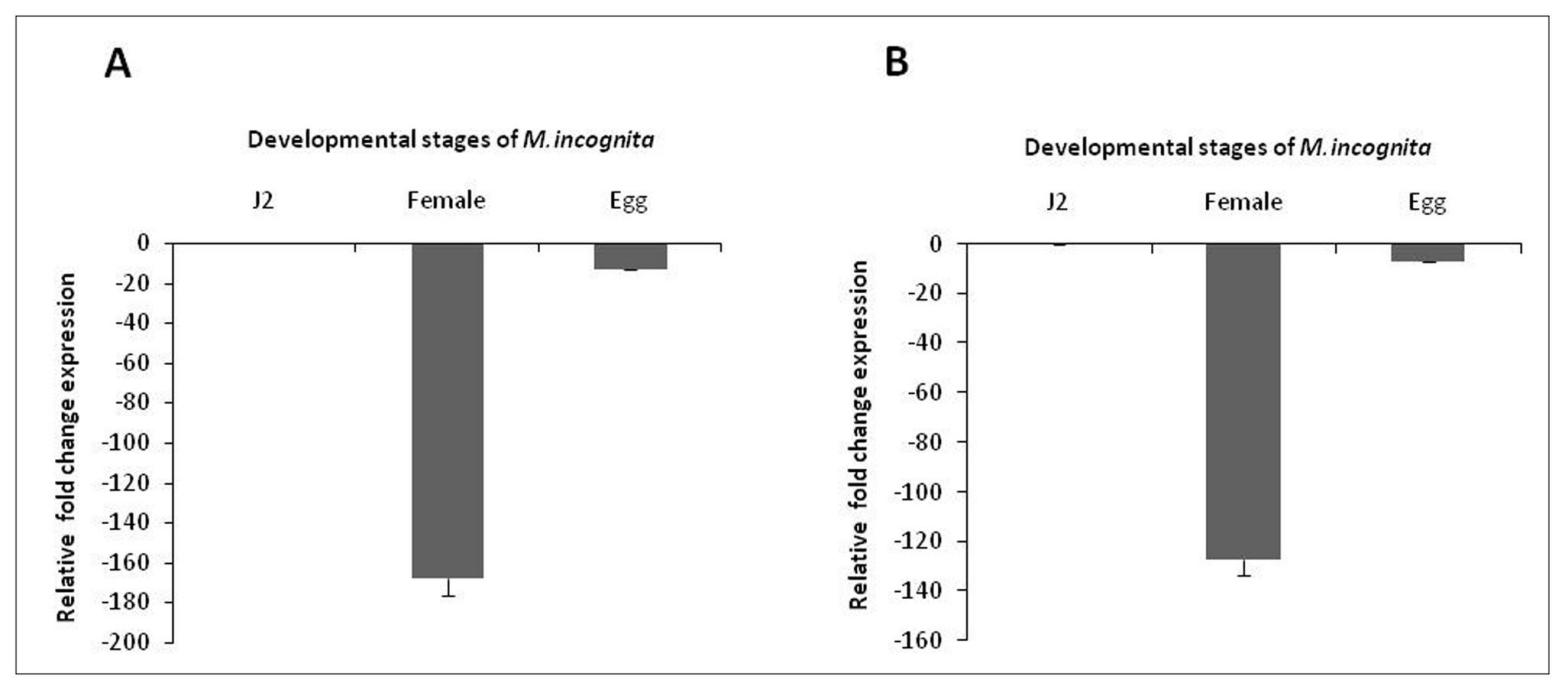
Relative fold change in the expression of target genes in different developmental stages of *Meloidogyne incognita*. (A) *flp-14* (B) *flp-18*. *18S* *rRNA* was used as a reference gene and fold change was calculated by using 2^-ΔΔCT^ method. Error bars show +SD among the biological replicates.

Cloning, sequencing and blast analysis of 284 bp and 353 bp (Figures S2A & S2B) of the coding sequences of *flp-14* and *flp-18* amplified from the cDNA of *M. incognita* revealed 100% similarity to the already reported sequences. Simultaneously, comparative expression of both the target genes by qRT-PCR in eggs, J2s and feeding females was carried out to analyze the spatial expression of both the genes. It was observed that the expression of both the genes was highest in J2s followed by eggs and females (Figure 1A & 1B). Relative fold expression of *flp-14* and *flp-18 was* reduced by about 170 and 130 times respectively in the females when compared to the J2s. However, expression of both the genes in the eggs had not reduced as much as in females. In eggs, there was a reduction of about 13 times for *flp-14* and 7 times for *flp-18* compared to the J2 stage. 

### *In vitro* RNAi by dsRNA soaking

**Figure 2:**
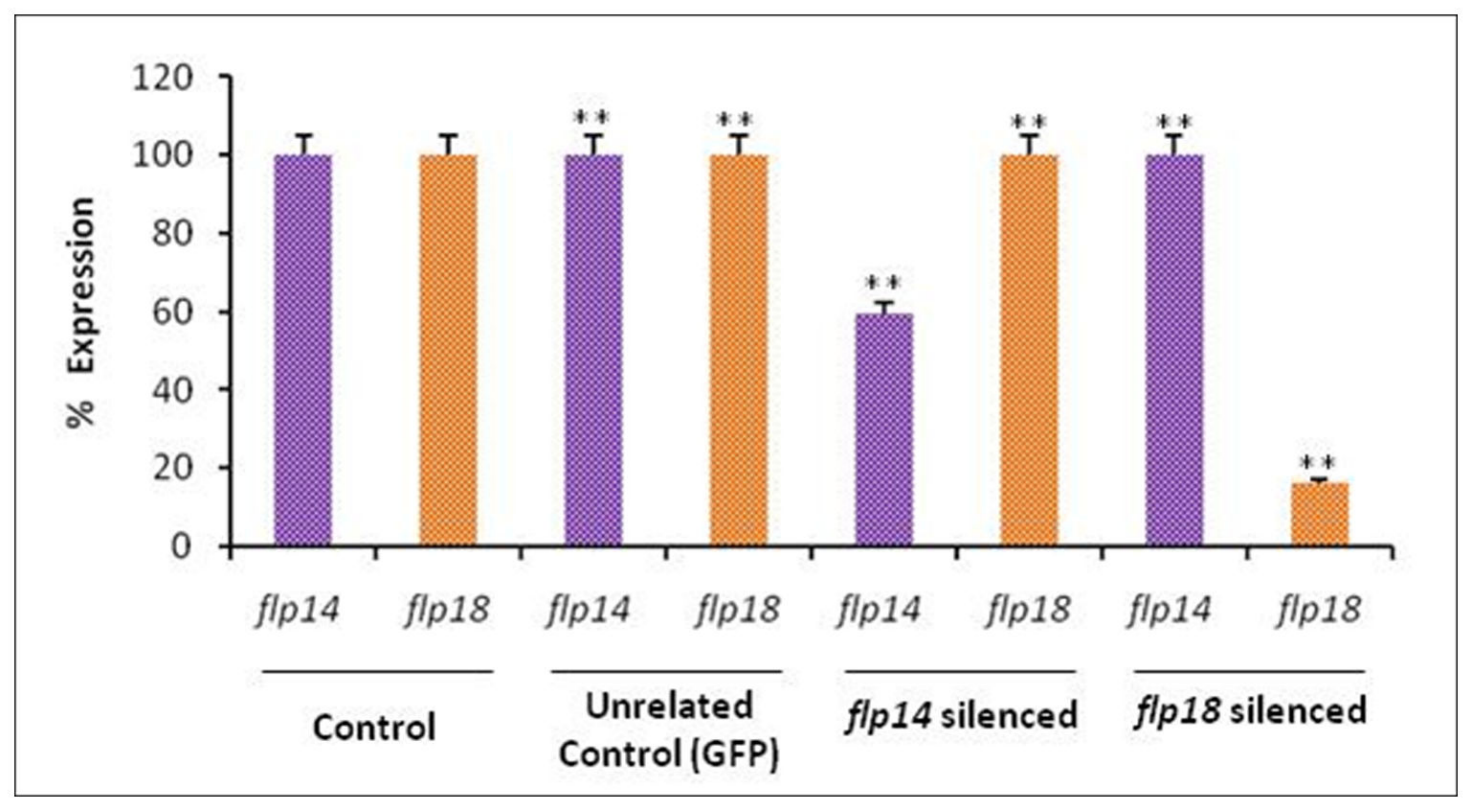
Effect of *in vitro* RNAi on transcript abundance of *flp-14* and *flp-18* in *Meloidogyne incognita* J2s. Expression was quantified to demonstrate target specific silencing in the nematodes. *18S* *rRNA* was used as reference gene and fold change was calculated by using 2^-ΔΔCT^ method. The fold change values were transformed to personate values. Error bars show +SD among the biological replicates. **P<0.01.

Initially, the effect of the cloned gene fragments on *M. incognita* was assessed by soaking in dsRNA of both the genes. As a first proof, silencing of the target genes, *flp-14* and *flp-18* was confirmed by qRT-PCR using cDNA obtained from the dsRNA soaked and control J2s. The transcript level was reduced in the dsRNA soaked J2s compared to the control worms (Figure 2). Silencing of *flp-14* resulted in about 40% transcript reduction while for *flp-18*, it was about 82%. In the present study, target specific silencing could be established using appropriate controls. Firstly, neither of the target genes were silenced in the GFP silenced worms (Figure 2). This was further substantiated by the absence of any adverse effect on *flp-18* in the *flp-14* silenced worms and vice versa (Figure 2). Expression level of reference gene, *18S rRNA*, was equal in both the treated and untreated nematodes. The stability of expression was confirmed in two biological replicates; each of which had three technical replications. This confirmed the production of siRNA leading to the silencing of the target genes.

**Figure 3:**
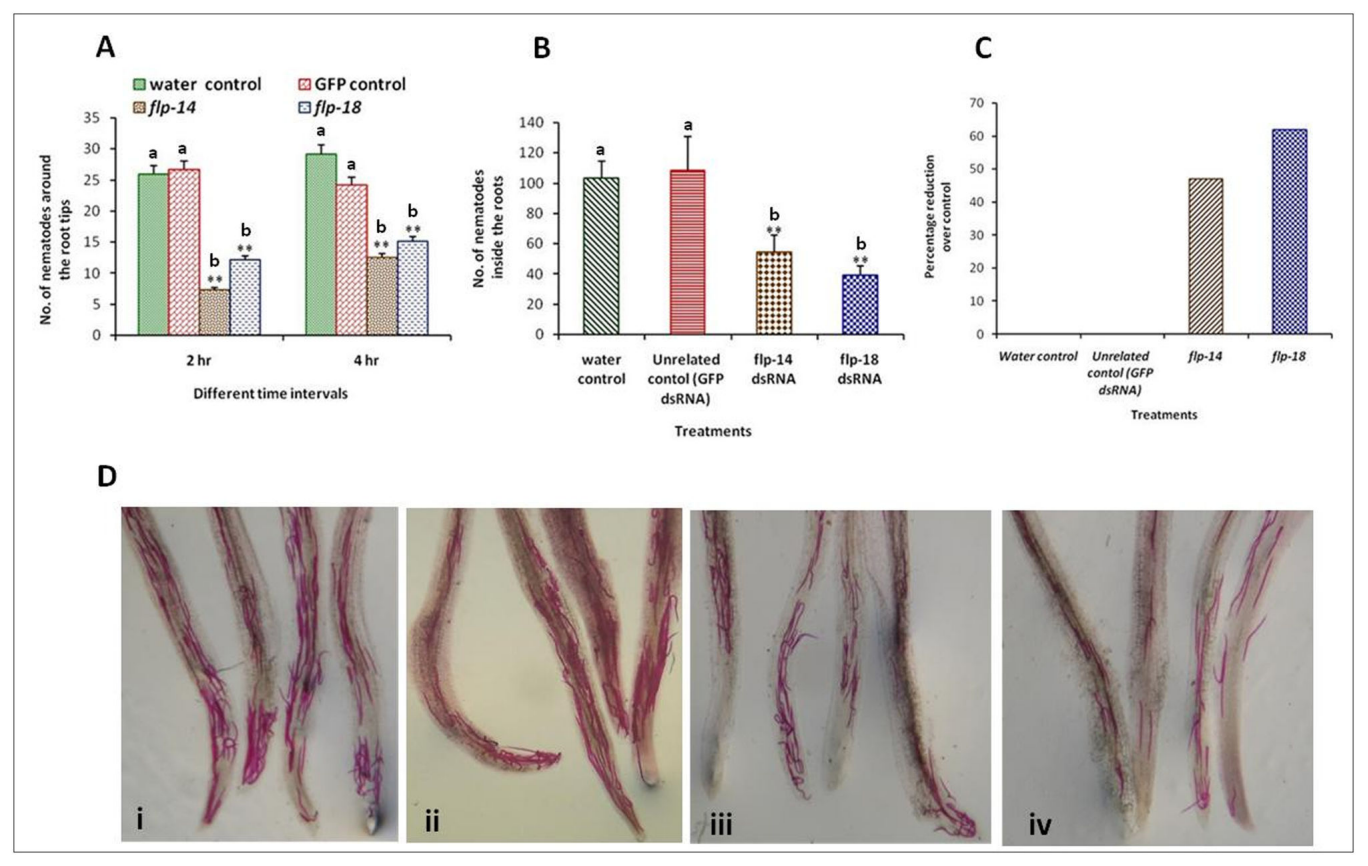
Effect of *in vitro* RNAi on nematode attraction and penetration. (A) Nematode attraction to tomato roots at different time intervals (B) Penetration into tomato roots at 24 h (C) Percentage reduction in nematode penetration in tomato roots due to RNAi compared to control (D) Stained nematodes in the infected tomato roots (i) Roots inoculated with J2s soaked in water (control) (ii) Roots inoculated with J2s soaked in dsRNA of GFP (Unrelated control) (iii) Roots inoculated with J2s soaked in dsRNA of *flp-14* (iv) Roots inoculated with J2s soaked in dsRNA of *flp-18*. Error bars show mean +SD of the number of J2s that could be seen within 0.5 cm diameter around or inside the root. Significant differences are marked with different alphabets, CRD test (_*_ P<0.05 and _**_ P< 0.01).

Subsequently, the phenotypic effect of gene silencing in terms of attraction and migration towards the host was assessed by an *in vitro* bioassay on pluronic gel. Corroborating with the expression analysis, the assay indicated reduced number of J2s reaching the roots. Silencing of both the target genes had a negative impact on the attraction and migration of the J2s and it was evident as early as 2 h and continued further even at 4 h (Figure 3A). The number of J2s reaching the roots reduced significantly at all the three intervals and by 24 h, most of the J2s reaching the roots had penetrated in both the treated and control worms (Figure 3B & 3C). In fact, the number of worms in the control was so high that the roots had to be dissected out to count the total number of J2s that had penetrated by 24 hrs while in treated worms, they could be counted without dissection (Figure 3D). Though the number of J2s migrating towards the roots were less in *flp-14* treated worms compared to *flp-18*, penetration was more in case of J2s silenced for *flp-14*. This indicated that silencing of *flp-18* was more effective than *flp-14* in reducing the penetration (Figure 3B). However, both the genes were validated *in planta* for the efficacy against *M. incognita*. This kind of adverse effect on nematode migration was not noticed when the worms were soaked in dsRNA of GFP confirming the target specific silencing and their negative effects on the nematode behavior.

### Validation studies of the RNAi vectors of *flp-14* and *flp-18* in tobacco using *Agrobacterium tumefaciens*

A very well standardized leaf-based regeneration system in tobacco was used to validate the efficacy of the hair pin RNAi constructs of *flp-14* and *flp-18*. The regenerated tobacco plants (T_0_ plants) containing the RNAi vectors of *flp-14* and *flp-18*, that could grow and establish on the selection medium containing 100 mg L^-1^ kanamycin were used for integration, expression and efficacy studies. 

### Analysis of the plants for T-DNA integration

Initially, 50 T_0_ plants each harbouring the dsRNA for *flp-14* and *flp-18* respectively were tested for the integration of T-DNA by PCR analysis. Analysis with different sets of primers amplifying the gene specific region, sense and the antisense region as well as the selectable markers demonstrated the amplification of the expected fragments in the transgenics with both *flp-14* and *flp-18* (Figure S3). This indicated the integration of the T-DNA in 40 out of 50 selected plants. 

**Figure 4:**
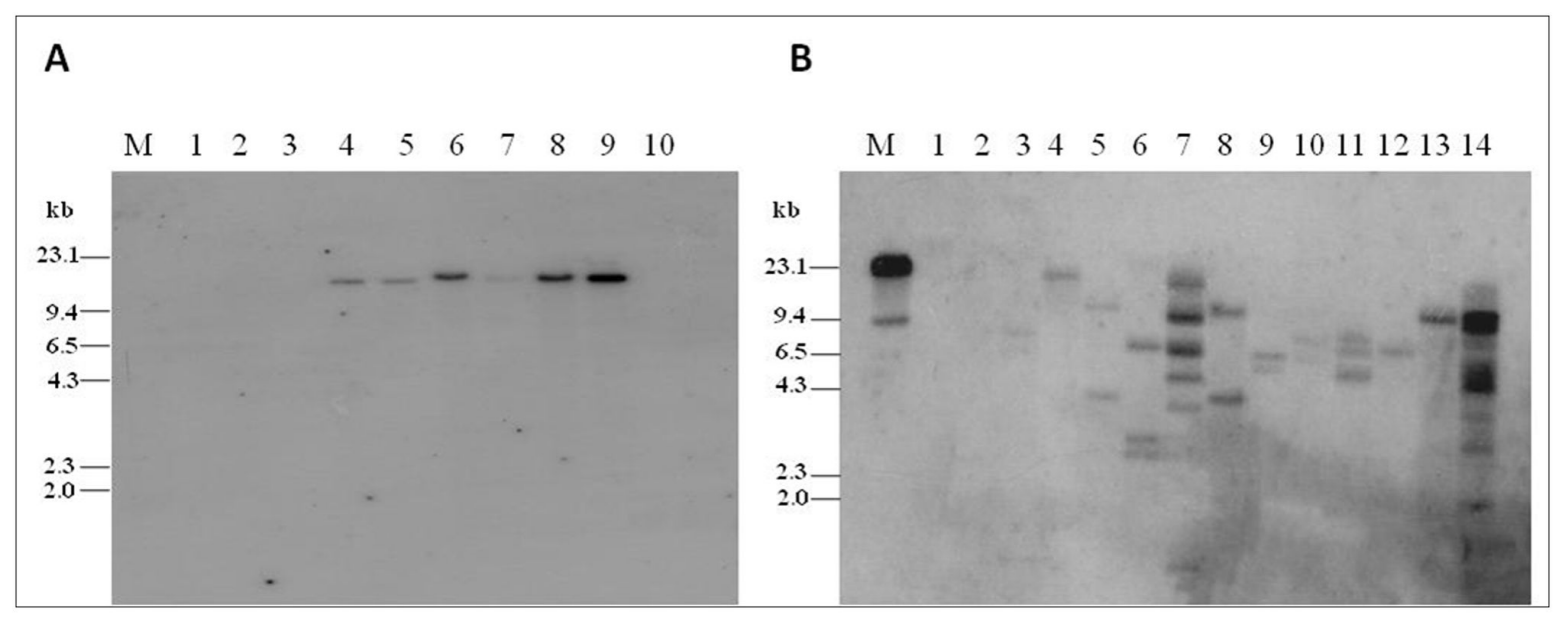
Genomic Southern analysis of the T_0_ transgenic events of tobacco harboring dsRNA of *flp-14* and *flp-18*. (A) Southern analysis to confirm the integration of *flp-14* gene. Lanes - M: Lambda *Hind*III digest, 1: Undigested wild type tobacco DNA, 2: Digested wild type tobacco DNA, 3-10: DNA samples from T_0_ transgenic tobacco events: 3- 133.1, 4-96.1, 5-44.2, 6-92.1, 7-52.1, 8-82.3, 9-95.3, 10-11.2 (B) Southern analysis to confirm the integration of *flp-18* gene. Lanes - M: Lambda Hind III digest, 1: Undigested wild type tobacco DNA, 2: Digested wild type tobacco DNA, 3-14: DNA samples from T_0_ transgenic tobacco events: 3- A.22, 4-A.6, 5-A.30, 6-A.31, 7-A.36, 8-A.37, 9-A.38, 10-A.39, 11-A.43, 12-A.45, 13-A.49, 14-A.55.

In order to confirm the integration pattern of the T-DNA, 8 events of *flp-14* and 12 events of *flp-18* were subjected to genomic southern analysis. Genomic DNA of all the T_0_ plants when digested with *Sac*I and probed with 284 and 353 bp fragments of the *flp-14* and *flp-18* genes respectively, revealed integration of the T-DNA in most of the events. There was no hybridization signal in the wild type plants used as negative control (Figure 4A & 4B). In the plants harbouring the hair pin RNAi vector for *flp-14*, all the plants showed single copy integration pattern (Figure 4A) demonstrating a clear integration of the T-DNA. Other set of plants harbouring RNAi vector of *flp-18* demonstrated a varied integration pattern in plants incorporating single and multiple copies of T-DNA (Figure 4B).

Based on the integration studies, 3 events harbouring *flp-14* (44.2, 82.3 and 92.1) and 6 events harbouring *flp-18* (A-6, A-30, A-36, A-38, A-39, A-45) were used for expression and efficacy analysis in the subsequent generation. The seeds of the selected plants were germinated and the T_1_ plants were maintained in the growth chamber for studies on expression and efficacy. However, as a preliminary proof of the inheritance of the T-DNA in the progeny of the selected events, PCR analysis was carried out in randomly selected T_1_ plants using primers for the amplification of the *flp-14, flp-18* gene specific region, sense and antisense regions and the selectable marker (Figure S4). It was observed that all the progeny plants showed the amplification of the expected fragments confirming the stable integration and inheritance of the T-DNA. 

### Validation of the progeny of the selected events for expression and efficacy of *flp-14* and *flp-18*

**Figure 5:**
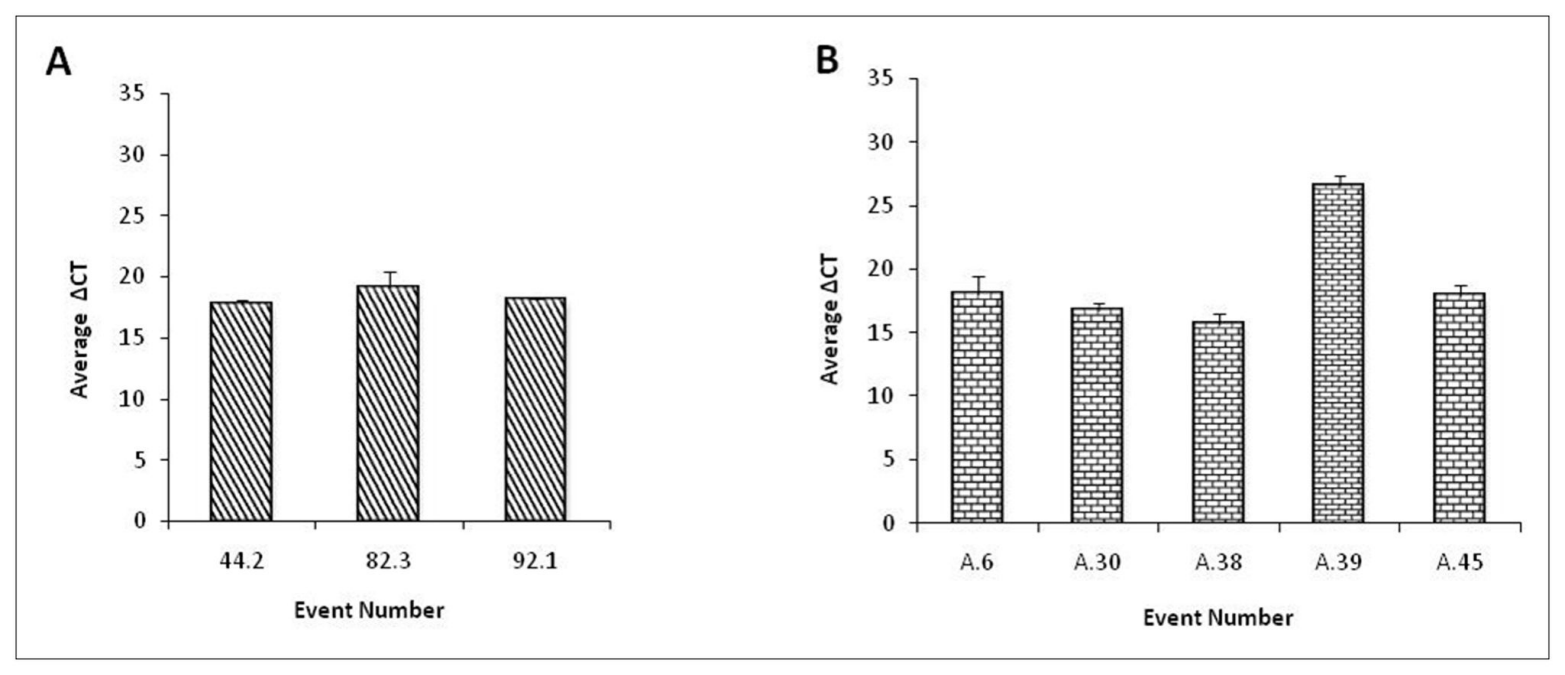
qRT-PCR analysis of T_1_ plants. Expression analysis of T_1_ transgenic tobacco plants (A) *flp-14* (B) *flp-18*. *18S* *rRNA* was used as a reference gene and ΔCT values were calculated using the difference in the ct mean of the target gene and reference gene. Error bars show +SD among the biological replicates.

**Figure 6:**
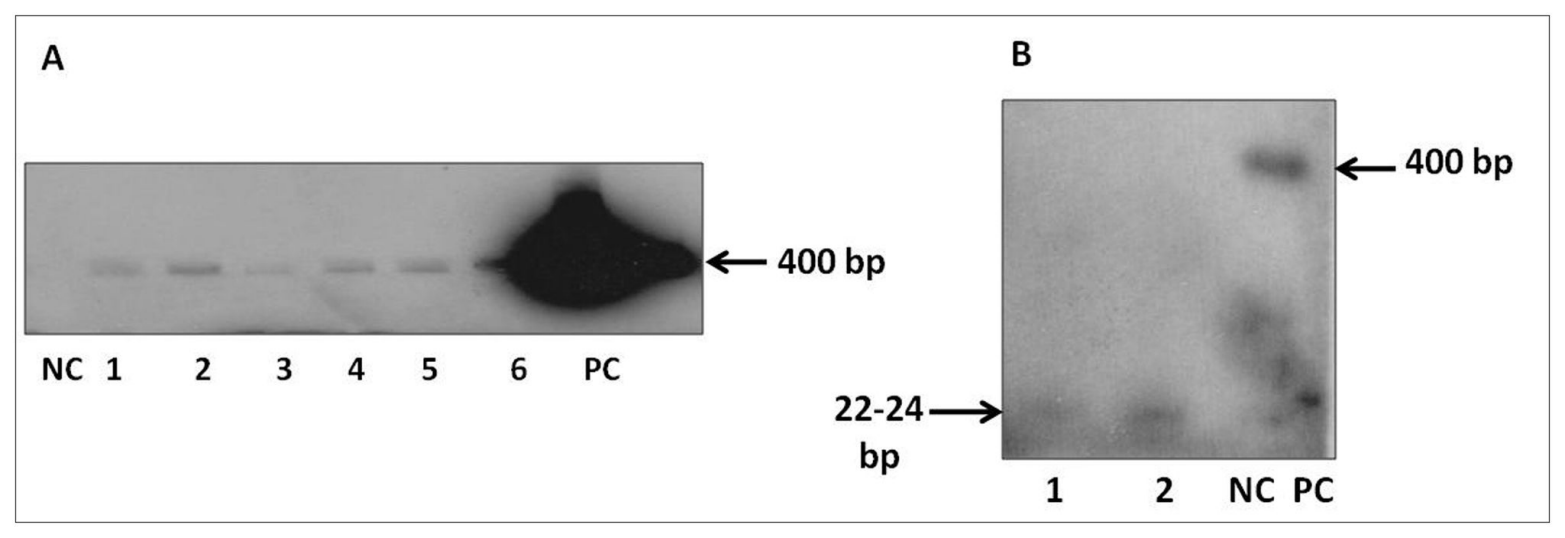
Confirmation of dsRNA and siRNA of *flp-18* in T_1_ plants by Northern analysis. (A) Northern blot for dsRNA; Lanes -NC: Negative control (Wild type tobacco plant), RNA samples from T_1_ transgenic tobacco events 1- A.6, 2- A.30, 3- A.38, 4- A.39, 5- A.45, 6 - A.43, PC: Positive control (Gene specific PCR product). (B) Northern blot for siRNA, Lanes 1&2: *flp-18* T_1_ tobacco events (A.45 & A.38), NC: Negative control (Wild type tobacco plant), PC: Positive control (Gene specific PCR product).

qRT-PCR analysis of the selected events was carried out to validate the expression of dsRNA transcript and its accumulation. Expression of the *flp-14* and *flp-18* dsRNA was confirmed in the progeny of the selected events. Six well established plants from each of the selected events were used for transcript analysis. It was observed that all the plants showed an increase in the transcript accumulation of *flp-14* when compared to the wild type (Figure 5A). However, there was variation amongst the lines that were chosen for the study and the average expression was higher in the event 44.2 compared to the other two events. Similarly, six well established and healthy plants belonging to each of the *flp-18* events evaluated were considered for expression analysis (Figure 5B). The event A-38 showed the highest expression of *flp-18* gene and the event A-39 showed the least. Additionally, as a key component of host-delivered RNAi, expression of dsRNA of *flp-18* was further established by Northern analysis in six transgenic plants comprising two plants for each of the three independent events which showed the presence of dsRNA in all the plants analyzed (Figure 6 A). Likewise, siRNA of *flp-18* could also be detected in the two representative samples (Figure 6B) confirming the possibility of host-delivered RNAi. 

### Bioefficacy of T_1_ tobacco lines against *M. incognita*

**Figure 7:**
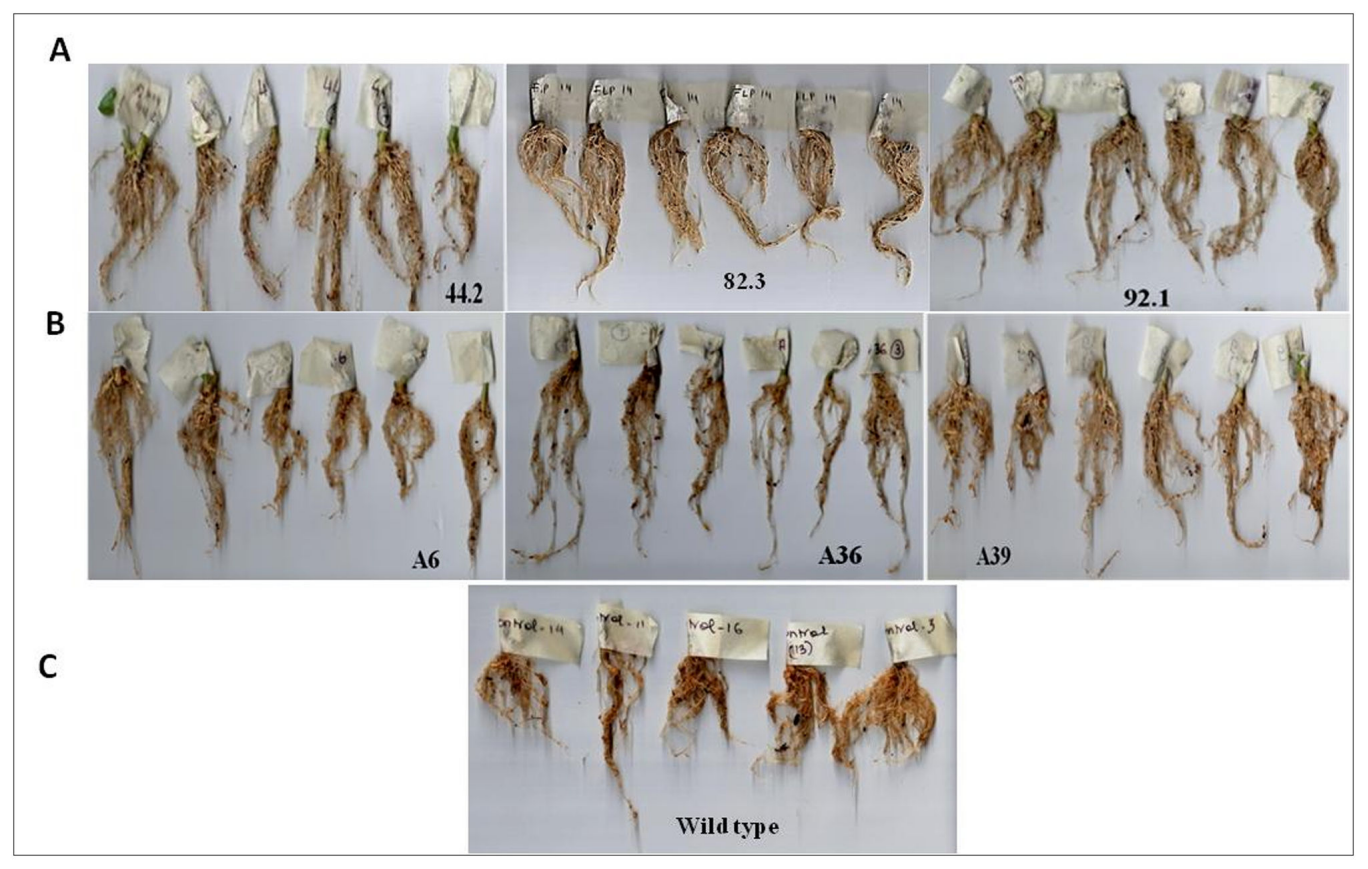
Bioefficacy analysis of the T_1_ tobacco events against *Meloidogyne incognita*. Intensity of galling in the roots of T_1_ tobacco plants expressing (A) *flp-14* (B) *flp-18* (C) Wild type.

**Figure 8:**
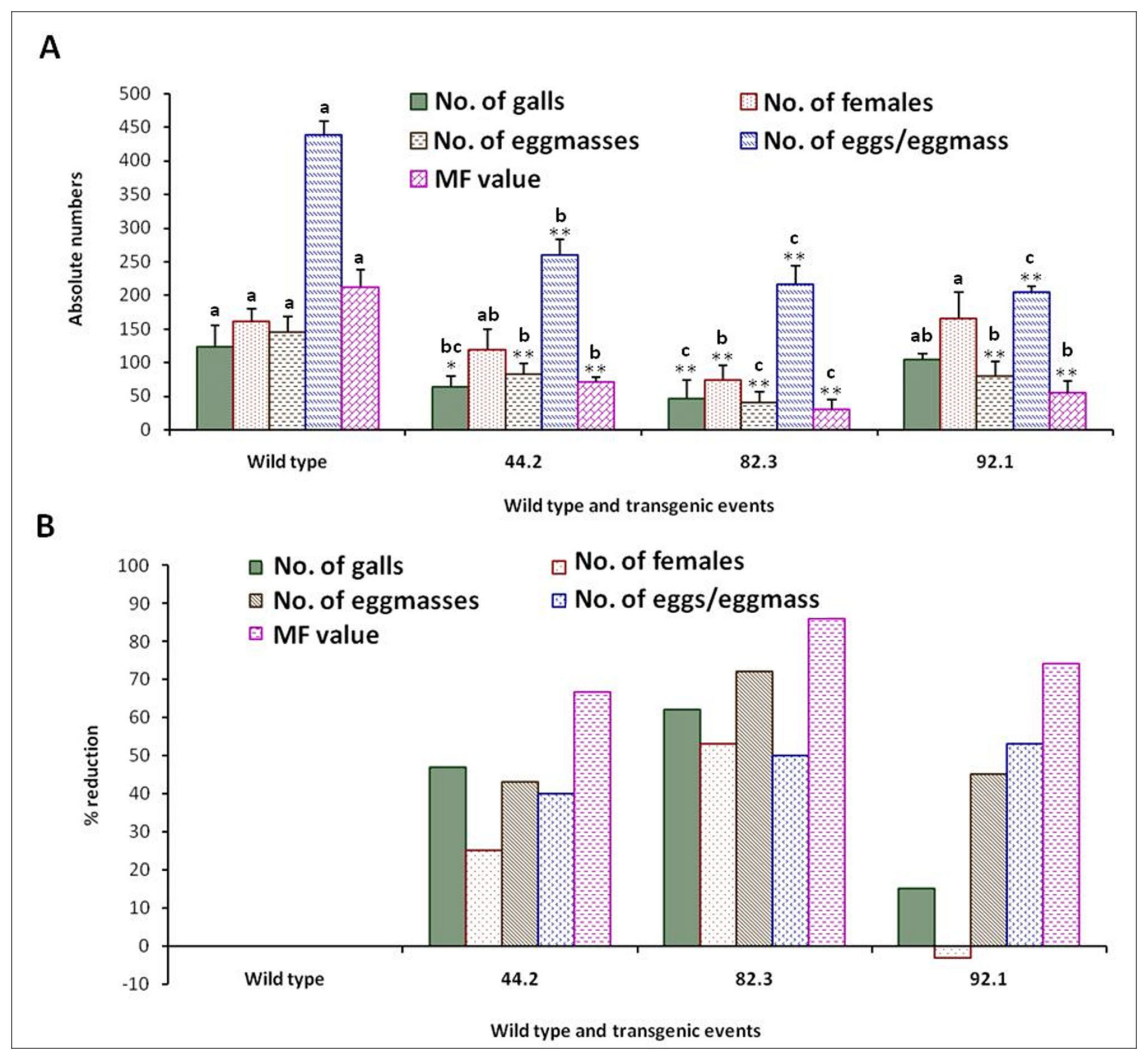
Effect of host delivered RNAi of *flp-14* on infection and reproduction of *Meloidogyne incognita*. (A) Infection and reproduction of *Meloidogyne incognita* (B) Percentage reduction in nematode infection and reproduction on transgenics compared to the wild type plants. Error bars show mean +SD. Significant differences are marked with different alphabets, CRD test (_*_ P<0.05 and _**_ P<0.01).

**Figure 9:**
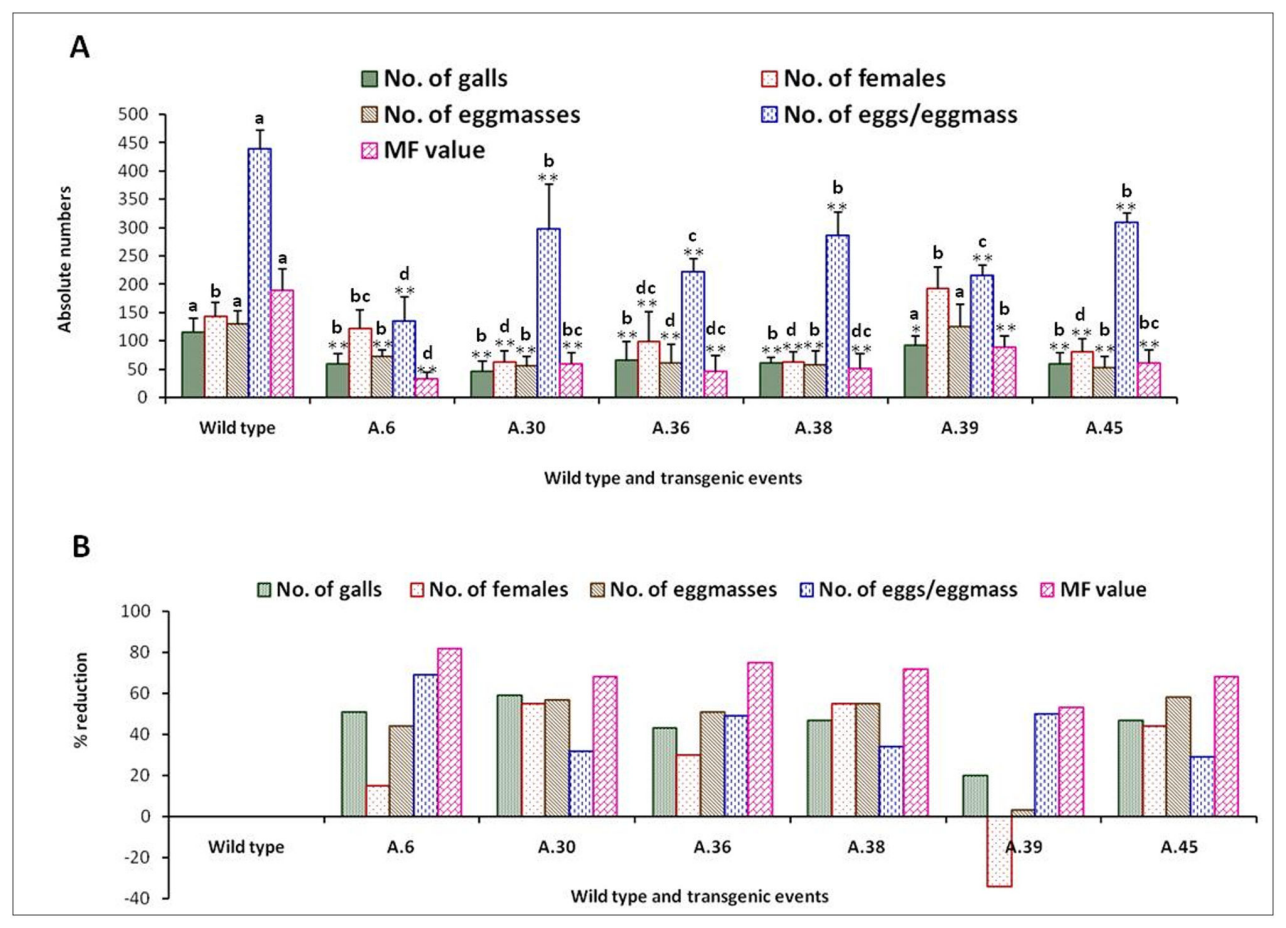
Effect of host delivered RNAi of *flp-18* on infection and reproduction of *Meloidogyne incognita*. (A) Reduction in infection and reproduction of *Meloidogyne incognita* due to silencing of *flp-18* (B) Percentage reduction in infection and reproduction of *M. incognita* on transgenics compared to wild type plants. Error bars show mean +SD. Significant differences are marked with different alphabets, CRD test (_*_ P<0.05 and _**_ P<0.01).

Nematode bioassays were performed in order to study the resistance offered against *M. incognita* by inoculating freshly hatched J2s on T_1_ generation plants that were confirmed for the *flp-14/flp-18* integration and expression of dsRNA. The plants were harvested 30 days after inoculation and observations were recorded on various parameters to determine the effect of host delivered RNAi on nematode infection, development and reproduction. T_1_ plants of the three selected independent events were screened in case of *flp-14* and the nematode infection was scored in terms of number of galls, females, egg masses and eggs per egg mass produced in each plant. Similarly, in case of transgenics for silencing the *flp-18* gene, T_1_ plants of six independent events were evaluated (Figure 7). It was quite evident that host delivered RNAi silencing of both the target genes resulted in reduced root galling due to the nematode infection and there was an increased root growth in the transgenics compared to the wild type plants. Silencing of both the genes generally reduced the nematode infection as indicated by reduction in the number of galls (Figures 8A & 9A). Correspondingly, there was a reduction in number of females and the percentage reduction ranged between 50 - 103 % in *flp-14* RNAi plants compared to the wild type plants. Interestingly, not all the females in these roots could reproduce since the percentage reduction of egg masses ranged from 28 to 57% which indicated that only 50% of the females could reproduce (Figure 8B). Likewise, silencing of *flp-14* reduced the fecundity of the nematodes as percentage reduction in number of eggs per egg mass ranged from 47 - 50 % (Figure 8B). Similarly, silencing of *flp-18* also exhibited such adverse effects on the nematode infection, development and reproduction (Figure 9A & 9B). Five out of the six events evaluated showed a reduction of 43 to 59 % in the total number of galls in the transgenic plants compared to the wild type. The reduction in number of females that developed per plant ranged from 15 to 50 %; reduction in fecundity reduced between 44-58%. While, that of eggs per egg mass was reduced between 29 and 69 percent. However, plants of one of the events (A39) expressing *flp-18* dsRNA were not effective in reducing the number of females and on the contrary more females were produced when compared to the wild type control plants. Surprisingly, the number of egg masses and the eggs per egg mass reduced even in this event resulting in 53% reduction in multiplication factor (MF) as compared to the wild type control roots. Finally, the nematode MF reflecting the overall ability of the nematode to be a successful parasite was reduced by about 67 to 86 % due to the silencing of *flp-14* while the reduction due to silencing of *flp-18* was 53 to 82%. 

**Figure 10:**
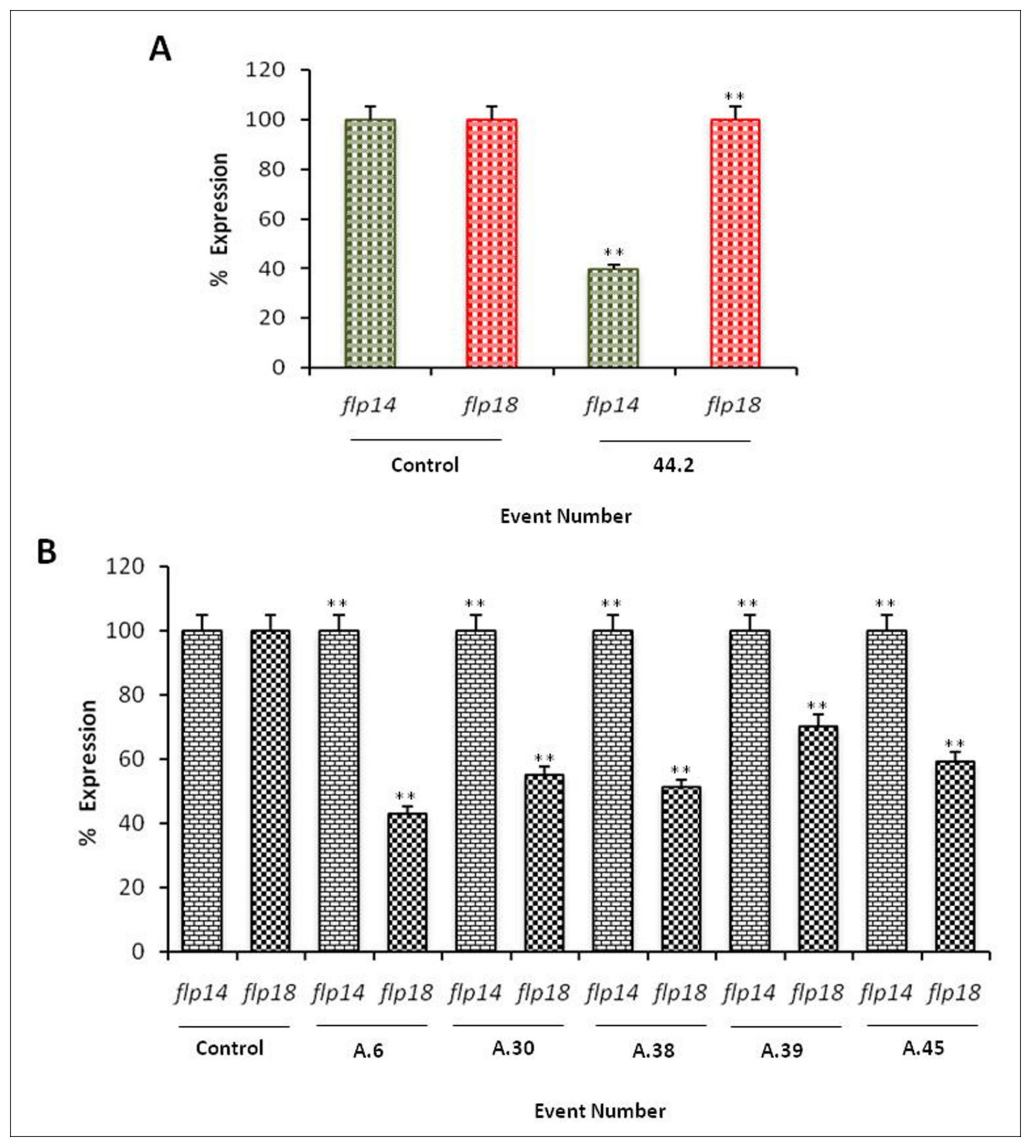
Percent reduction in the relative transcript levels due to target gene silencing in the females of *M*. *incogntia* feeding on the transgenic plants. (A) Decrease in mRNA abundance in *M. incogntia* females extracted from (A) *flp-14* (B) *flp-18* transgenic tobacco lines. Expression was quantified to demonstrate the target specific gene silencing in the nematodes through host delivered RNAi of *flp-14* and *flp-18*. *18S* *rRNA* was used as a reference gene and fold change was calculated by using 2^-ΔΔCT^ method. Fold change values were transformed to personate values. Error bars show +SD among the biological replicates. ** P<0.01.

An interesting corroboration with the bioefficacy was observed by qRT-PCR quantification of *flp-14* and *flp-18* genes (Figure 10A & 10B) in the females extracted from the transgenic tobacco plants harboring the respective genes. The observation showed down regulation of the target genes suggesting effective host delivered RNAi in the transgenic plants. Target specific host delivered gene silencing could also be established as in case of *in vitro* RNAi silencing. No reduction in the quantification of *flp-18* transcript was observed in the females extracted from the transgenic plants expressing dsRNA of *flp-14* and vice versa confirming the target specific gene silencing by *in planta*. 

## Discussion

Neuropeptides, particularly FLPs, are associated with probably all physiological systems in nematodes that include sensory systems, feeding, locomotion and reproduction essential for successful parasitism. Therefore, interference of neuropeptides or FLPs can lead to disruption of multiple functions. In view of this, they could be potential targets for crop improvement against nematodes. The existing information on neurobiology of *C. elegans* and *A.suum* is very valuable to identify the suitable *flp* genes for the control of economically important species of root knot nematodes, *M. incognita* [10,35]. To date, 19 *flp* genes have been reported in *M. incognita* [1]. In this direction, focus of the present study was mainly on the validation of two *flp* genes, *flp-14* and *flp-18* for *M. incognita* parasitism through host-delivered RNAi in tobacco. 

The expression pattern of the genes in three developmental stages demonstrated importance of the target genes in both eggs and infective larval stages. Further, the emphasis was to demonstrate the utility of the cloned genes as suitable targets for *in vitro* and host-derived RNAi. An unrelated gene, GFP, was used as a negative control to prove the target specific gene silencing during *in vitro* evaluation.

*In vitro* RNAi of a nematode gene could provide validation for host delivered RNAi of that gene. It has been well established in *G. pallida* [17], *G. rostochiensis* [36], *Heterodera schactii* [37], *H. glycines* [17,38-41], *M. incognita* [19,21,22,24,42], *M. javanica* [43,44] and *Pratylechus* spp [45]. In the present study, *in vitro* RNAi of both *flp-14* and *flp-18* revealed silencing that was adequately supported by penetration assays and expression analysis.

Silencing of *flp-14* reduced the migration as well as the penetration of *M. incogntia* J2s. This could be corroborated by transcript quantification that revealed 40% less expression in *flp-14* silenced J2s compared to the control worms. Localization of *flp-14* in *M. incognita* indicated its expression in the four SMB-like neurons posterior to the central nerve ring [46]. In *C. elegans* the SMBs connect indirectly with amphid (sensory) and motor neurons suggesting their role in locomotion and sensory perception. This supports the reduced migration and penetration observed in our study as *flp-14* silencing could have interfered with the sensory perception which in turn affected the respective motor function in the dsRNA soaked worms. Similar effects were observed when *flp-14* was silenced in *G. pallida* by soaking in dsRNA for 24 h [18]. Correspondingly, silencing of *flp-18* resulted in 84 % transcript knock down in the treated J2s when compared to the control worms. No effect on *flp-14* was observed due to the silencing of *flp-18* and vice versa indicating target specific gene silencing which was also supported by the absence of any such effects in the GFP silenced worms.

Knockdown of *flp-18* resulted in reduced migration and penetration of the treated J2s when compared to the control worms reflecting the physiological effect. Earlier reports also showed reduced migration of J2s due to the silencing of *flp-18* in *M. incognita* [15]. Similar effects were also observed in *G. pallida* when *flp-18* was silenced resulting in almost complete inhibition of migratory behavior after 24 hr incubation in dsRNA [18]. *Flp-18* is expressed in the neurons that synapse directly onto the pharyngeal muscles and thereby control their action [47]. Localization studies in *C. elegans* using *flp-18* reporter gene constructs revealed its expression in the specific interneurons AVA, AIY and RIG, the motor neuron RIM and pharyngeal neurons M2 and M3 [48]. The AIY neurons are postsynaptic to olfactory, gustatory and thermosensory neurons [49]. *Flp-18* mutants in *C. elegans* were defective in chemosensation, foraging, dauer formation, fat accumulation and also exhibited decreased oxygen consumption [50]. AIY neurons also regulate another food related behavior called local search. When wild type animals are removed from food, they explore a local area, where food is expected, by repeatedly reversing and turning. After about 15 min of food withdrawal, animals switch to exploring larger areas, by inhibiting reversals and turns. Animals lacking the AIY interneurons continue reversing and turning even an hour after removal from food [51-53]. Further release of *flp-18* from AIY neurons regulates odor responses, foraging ability and fat metabolism. Since M2 and M3 synapse directly onto the pharyngeal muscle [47], they are responsible for modulation of pharyngeal activity. Ablation of M3 and two other neurons in the larvae of *C. elegans* lead to changes in feeding behavior and a decline in growth rate [54,55]. Based on these observations and understanding about the *flp-18* functions, the observed reduction in migration and penetration could be attributed to the knock down effect of *flp-18*. The observed phenotypic effects in the plant root growth coupled with the reduction in the nematode infection could be attributed to the reduction in transcripts of *flp-14* and *flp-18* due to the uptake of dsRNA by the nematodes. Therefore, *in vitro* RNAi demonstrated the requirement and importance of *flp-14* and *flp-18* for normal sensory perception and mobility. 

On confirming the RNAi silencing of *flp-14* and *flp-18* by *in vitro* studies, additional proof for the silencing of the two target genes and subsequent effect on the nematode was undertaken by *in planta* validation using a highly efficient tobacco system. Several molecular analyses demonstrated the integration and inheritance of the T-DNA harboring the dsRNA hairpin construct of both the genes independently. However, more important would be to know how efficiently the highly expressing plants would resist the attack of nematodes. Therefore, the bioefficacy studies would be of prime importance. Stringent observations of the challenged plants did not show any phenotypic variations indicating the focused effect of the RNAi. The selected transgenic plants showed remarkable protection against the nematodes as there was significant reduction in number of galls, females, egg masses etc. indicating the efficacy of the RNAi silencing that interrupted the sensory perception and motor functions. These observations corroborated with the expression analysis as the plants that showed high expression could resist nematode infection better and ultimately resulted in reduced reproduction.

The presence of the dsRNA and the siRNA in the transgenic plants provided the ultimate evidence for the host delivered RNAi of *flp-18* that resulted in reduced nematode reproduction*.*

The present findings are similar to the earlier report involving injection of FMRFamide related peptides of *flp-14* and 15 other *flps* that exhibited inhibitory actions on the ovijector in *A. suum* [56]. These effects generally include shortening of the oviduct, consistent with circular muscle relaxation, and a cessation of contractile activity that could affect the egg laying ability of the nematodes. This could be the cause for reduced reproduction in the worms infesting the transgenic tobacco lines expressing *flp-14* and *flp-18* dsRNA. The response in the bioeffeicacy studies clearly corroborated with the finding of *in vitro* RNAi emphasizing the need of the selected genes for important functions of the nematode leading to successful parasitism.

However, the response to the nematode could depend on a threshold level of transgene expression leading to effective silencing. For this, one of the *flp-18* events, A-39.1 was chosen. The event showed very low expression in the qRT-PCR analysis. As supporting evidence, it was observed that the event did not demonstrate bioefficacy as comparable with the other highly expressing events and the corresponding percent reduction of the galls etc was not profound. This clearly showed the requirement of a threshold level of dsRNA expression in the host for the silencing to occur effectively. Similar observation was also seen when the extent of silencing of the gene was quantified by qRT-PCR in the nematodes extracted from the transgenic tobacco plants that were used in the bioefficacy studies. This kind of varied expression levels has not been reported in other host delivered RNAi studies. 

The results were quite explicit in demonstrating the possibility of host delivered RNAi of the two important FLP genes independently for the management of *M. incognita* on tobacco. These findings have been adequately supported by the inclusion of negative controls during both *in vitro* and *in planta* RNAi experiments. Thus, the observed phenotypic effects can be unequivocally attributed to the target specific gene silencing.

Though there are many reports of utility of *flps* for disrupting the neuromotor function, this is the first established report of host delivered RNAi of *flp-14* and *flp-18* for the management of *M. incognita*. The study effectively substantiated that the reduction in nematode multiplication factor was due to the silencing of *flp-14* and *flp-18*. Therefore, it reiterates that the two genes would be of immense value to reduce the initial population densities in the field which in turn could bring down the resident population pressure in the soil for the subsequent crop. This established a proof of concept for using FLP-based RNAi transgenics for the management of *M. incognita*. Further, it opens options for stacking more than one *flp* gene to disrupt different functions more efficiently so as to achieve durable management. In addition, the genes and the strategy can also be effectively extended into improvement programs of other crops where the nematode is a serious concern. 

## Supporting Information

Figure S1 **Hair pin RNAi constructs of *flp-14* and *flp-18* used for validation studies in tobacco.**
(TIF)

Figure S2 **PCR amplification of *flp-14* and *flp-18* and confirmation of cloning by restriction digestion.** (A) Amplification of the target genes from cDNA of *Meloidogyne incognita* Lanes - 1: 100 bp DNA Ladder, 2: *flp-14* (284 bp), 3: *flp-18* (407 bp). (B) Confirmation of the inserts in the recombinant pGEM-T vector by EcoRI digestion. Lanes - 1: 100 bp DNA Ladder, 2: Undigested plasmid, 3: *flp-14* (284 bp), 4: *flp-18* (407 bp).
(TIF)

Figure S3 **PCR analysis of T_0_ transgenic tobacco plants expressing dsRNA of *flp-14* and *flp-18*.** (A) Amplification of the target genes from T_0_ transgenic plants using gene specific primers. Lanes - 1: 100 bp DNA Ladder, 2-20: Primary events of *flp-14* (342 bp), *flp-18* (411 bp) (B) Amplification of sense strand in T_0_ transgenic events using primers to 35S promoter and *attb2*. Lanes - 1: 100 bp DNA Ladder, 2-20: Primary events of *flp-14* (477 bp), *flp-18* (546 bp). (C) Amplification of the antisense strand in T_0_ transgenic events using primers to amplify 35S terminator and *attb2*. Lanes - 1: 100 bp DNA Ladder, 2-20: Primary events of *flp-14* (441 bp), *flp-18* (510 bp). (D) Amplification of *nptII* gene from primary transgenic events. Lanes - 1: 100 bp DNA Ladder, 2-20: T_0_ events (750 bp).
(TIF)

Figure S4 **PCR analysis of T_1_ plants expressing dsRNA of *flp-14* and *flp-18*.** (A) Amplification of the target genes from T_1_ plants using gene specific primers. Lanes - 1: 100 bp DNA Ladder, 2-20: different T_1_ plants of *flp-14* (342 bp), *flp-18* (411 bp) (B) 35S promoter and the target gene fragment. Lanes - 1: 100 bp DNA Ladder, 2-20: different T_1_ events of *flp-14* (477 bp), *flp-18* (546 bp). (C) 35S terminator and target gene fragment from T_1_ transgenic events Lanes - 1: 100 bp DNA Ladder, 2-20: different T_1_ events of *flp-14* (441 bp), *flp-18* (510 bp). (D) *npt*II gene fragment. Lanes - 1: 100 bp DNA Ladder, 2-20: different T_1_ events (750 bp).
(TIF)

## References

[1] Abad P, Gouzy J, Aury JM, Castagnone-Sereno P, Danchin EG et al. (2008) Genome sequence of the metazoan plant-parasitic nematode Meloidogyne incognita. Nat Biotechnol 26: 909-915. doi:10.1038/nbt.1482. PubMed: 18660804. 18660804

[2] Sasser JN (1980) Root-knot nematodes: A global menace to cropproduction. Plant Dis 64: 34-41.

[3] Ibrahim HM, Alkharouf NW, Meyer SL, Aly MA, Gamal El-Din Ael K et al. (2011) Post-transcriptional gene silencing of root-knot nematode in transformed soybean roots. Exp Parasitol 127: 90-99. doi:10.1016/j.exppara.2010.06.037. PubMed: 20599433. 20599433

[4] Trudgill DL, Blok VC (2001) Apomictic, polyphagous root-knot nematodes: exceptionally successful and damaging biotrophic root pathogens. Annu Rev Phytopathol 39: 53-77. doi:10.1146/annurev.phyto.39.1.53. PubMed: 11701859. 11701859

[5] Day TA, Maule AG (1999) Parasitic peptides! The structure and function of neuropeptides in parasitic worms. Peptides 20: 999-1019. doi:10.1016/S0196-9781(99)00093-5. PubMed: 10503780. 10503780

[6] Geary TG, Marks NJ, Maule AG, Bowman JW, Alexander-Bowman SJ et al. (1999) Pharmacology of FMRFamide-related peptides in helminths. Ann N Y Acad Sci 897: 212-227. doi:10.1111/j.1749-6632.1999.tb07893.x. PubMed: 10676450. 10676450

[7] Brownlee DJ, Fairweather I, Holden-Dye L, Walker RJ (1996) Nematode neuropeptides: Localization, isolation and functions. Parasitol Today 12: 343-351. doi:10.1016/0169-4758(96)10052-1. PubMed: 15275172. 15275172

[8] Fellowes RA, Maule AG, Marks NJ, Geary TG, Thompson DP et al. (1998) Modulation of the motility of the vagina vera of Ascaris suum in vitro by FMRF amide-related peptides. Parasitology 116 (3): 277-287. doi:10.1017/S0031182097002229. PubMed: 9550221. 9550221

[9] Fellowes RA, Maule AG, Martin RJ, Geary TG, Thompson DP et al. (2000) Classical neurotransmitters in the ovijector of Ascaris suum: localization and modulation of muscle activity. Parasitology 121 ( 3): 325-336. doi:10.1017/S0031182099006290. PubMed: 11085252. 11085252

[10] McVeigh P, Geary TG, Marks NJ, Maule AG (2006) The FLP-side of nematodes. Trends Parasitol 22: 385-396. doi:10.1016/j.pt.2006.06.010. PubMed: 16824799. 16824799

[11] Maule AG, Mousley A, Marks NJ, Day TA, Thompson DP et al. (2002) Neuropeptide signaling systems - potential drug targets for parasite and pest control. Curr Top Med Chem 2: 733-758. doi:10.2174/1568026023393697. PubMed: 12052188. 12052188

[12] Mousley A, Moffett CL, Duve H, Thorpe A, Halton DW et al. (2005) Expression and bioactivity of allatostatin-like neuropeptides in helminths. Int J Parasitol 35: 1557-1567. doi:10.1016/j.ijpara.2005.08.002. PubMed: 16185693. 16185693

[13] Kimber MJ, Fleming CC, Bjourson AJ, Halton DW, Maule AG (2001) FMRFamide-related peptides in potato cyst nematodes. Mol Biochem Parasitol 116: 199-208. doi:10.1016/S0166-6851(01)00323-1. PubMed: 11522352. 11522352

[14] Xue B, Hamamouch N, Li C, Huang G, Hussey RS et al. (2013) The 8D05 parasitism gene of Meloidogyne incognita is required for successful infection of host roots. Phytopathology 103: 175-181. doi:10.1094/PHYTO-07-12-0173-R. PubMed: 23294405. 23294405

[15] Dalzell JJ, McMaster S, Fleming CC, Maule AG (2010) Short interfering RNA-mediated gene silencing in Globodera pallida and Meloidogyne incognita infective stage juveniles. Int J Parasitol 40: 91-100. doi:10.1016/j.ijpara.2009.07.003. PubMed: 19651131. 19651131

[16] Dalzell JJ, McMaster S, Johnston MJ, Kerr R, Fleming CC et al. (2009) Non-nematode-derived double-stranded RNAs induce profound phenotypic changes in Meloidogyne incognita and Globodera pallida infective juveniles. Int J Parasitol 39: 1503-1516. doi:10.1016/j.ijpara.2009.05.006. PubMed: 19482028. 19482028

[17] Urwin PE, Lilley CJ, Atkinson HJ (2002) Ingestion of double-stranded RNA by preparasitic juvenile cyst nematodes leads to RNA interference. Mol Plant Microbe Interact 15: 747-752. doi:10.1094/MPMI.2002.15.8.747. PubMed: 12182331. 12182331

[18] Kimber MJ, McKinney S, McMaster S, Day TA, Fleming CC et al. (2007) flp gene disruption in a parasitic nematode reveals motor dysfunction and unusual neuronal sensitivity to RNA interference. FASEB J 21: 1233-1243. doi:10.1096/fj.06-7343com. PubMed: 17200420. 17200420

[19] Bakhetia M, Charlton WL, Urwin PE, McPherson MJ, Atkinson HJ (2005) RNA interference and plant parasitic nematodes. Trends Plant Sci 10: 362-367. doi:10.1016/j.tplants.2005.06.007. PubMed: 16027029. 16027029

[20] Shingles J, Lilley CJ, Atkinson HJ, Urwin PE (2007) Meloidogyne incognita: molecular and biochemical characterisation of a cathepsin L cysteine proteinase and the effect on parasitism following RNAi. Exp Parasitol 115: 114-120. doi:10.1016/j.exppara.2006.07.008. PubMed: 16996059. 16996059

[21] Rosso MN, Dubrana MP, Cimbolini N, Jaubert S, Abad P (2005) Application of RNA interference to root-knot nematode genes encoding esophageal gland proteins. Mol Plant Microbe Interact 18: 615-620. doi:10.1094/MPMI-18-0615. PubMed: 16042006. 16042006

[22] Niu JH, Jian H, Xu JM, Chen CD, Guo QX et al. (2012) RNAi silencing of the Meloidogyne incognita Rpn7 gene reduces nematode parasitic success. Eur J Plant Pathol 134: 131-144. doi:10.1007/s10658-012-9971-y.

[23] Johnathan JD, Steven M, Michael J, Rachel K, Colin C et al. (2009) Non-nematode-derived double-stranded RNAs induce profound phenotypic changes in *Meloidogyne* *incognita* and *Globoderapallida* infective juveniles. Int J Parasitol 39: 1503–1516. doi:10.1016/j.ijpara.2009.05.006. PubMed: 19482028. 19482028

[24] Huang G, Allen R, Davis EL, Baum TJ, Hussey RS (2006) Engineering broad root-knot resistance in transgenic plants by RNAi silencing of a conserved and essential root-knot nematode parasitism gene. Proc Natl Acad Sci U S A 103: 14302-14306. doi:10.1073/pnas.0604698103. PubMed: 16985000. 16985000 PMC1570184

[25] Yadav BC, Veluthambi K, Subramaniam K (2006) Host-generated double stranded RNA induces RNAi in plant-parasitic nematodes and protects the host from infection. Mol Biochem Parasitol 148: 219-222. doi:10.1016/j.molbiopara.2006.03.013. PubMed: 16678282. 16678282

[26] Fairbairn DJ, Cavallaro AS, Bernard M, Mahalinga-Iyer J, Graham MW et al. (2007) Host-delivered RNAi: an effective strategy to silence genes in plant parasitic nematodes. Planta 226: 1525-1533. doi:10.1007/s00425-007-0588-x. PubMed: 17653759. 17653759

[27] Steeves RM, Todd TC, Essig JS, Trick HN (2006) Transgenic soybeans expressing siRNAs specific to a major sperm protein gene suppress Heterodera glycines reproduction. Funct Plant Biol 33: 991–999. doi:10.1071/FP06130. 32689310

[28] Sindhu AS, Maier TR, Mitchum MG, Hussey RS, Davis EL et al. (2009) Effective and specific in planta RNAi in cyst nematodes: expression interference of four parasitism genes reduces parasitic success. J Exp Bot 60: 315-324. PubMed: 19015219. 19015219 10.1093/jxb/ern289 PMC3071771

[29] Gantasala NP, Papolu PK, Thakur PK, Kamaraju D, Sreevathsa R et al. (2013) Selection and validation of reference genes for quantitative gene expression studies by real-time PCR in eggplant (Solanum melongena L). BMC Res Notes 6: 312. doi:10.1186/1756-0500-6-312. PubMed: 23919495. 23919495 PMC3750715

[30] Livak KJ, Schmittgen TD (2001) Analysis of relative gene expression data using real-time quantitative PCR and the 2(-Delta Delta C(T)) Method. Methods 25: 402-408. doi:10.1006/meth.2001.1262. PubMed: 11846609. 11846609

[31] Wang CL, Lower S, Williamson VM (2009) Application of Pluronic gel to the study of root-knot nematode behaviour. Nematology 11: 453-464. doi:10.1163/156854109X447024.

[32] Bybd DW, Kirkpatrick T, Barker KR (1983) An improved technique for clearing and staining plant tissues for detection of nematodes. J Nematol 15: 142-143. PubMed: 19295781. 19295781 PMC2618249

[33] Karimi M, Inzé D, Depicker A (2002) GATEWAY vectors for Agrobacterium-mediated plant transformation. Trends Plant Sci 7: 193-195. doi:10.1016/S1360-1385(02)02251-3. PubMed: 11992820. 11992820

[34] Southern EM (1975) Detection of specific sequences among DNA fragments separated by gel electrophoresis. J Mol Biol 98: 503-517. doi:10.1016/S0022-2836(75)80083-0. PubMed: 1195397. 1195397

[35] Li C, Nelson LS, Kim K, Nathoo A, Hart AC (1999) Neuropeptide gene families in the nematode Caenorhabditis elegans. Ann N Y Acad Sci 897: 239-252. doi:10.1111/j.1749-6632.1999.tb07895.x. PubMed: 10676452. 10676452

[36] Chen PC, Roberts PA (2003) Genetic analysis of (a)virulence in Meloidogyne hapla to resistance in bean (Phaseolus vulgaris). Nematology 5: 687-697. doi:10.1163/156854103322746869.

[37] Vanholme B, Mitreva M, Van Criekinge W, Logghe M, Bird D et al. (2005) Detection of putative secreted proteins in the plant-parasitic nematode *Heterodera* *schachtii* . Parasitol Res 98: 414-424. PubMed: 16380840. 16380840 10.1007/s00436-005-0029-3

[38] Lilley CJ, Goodchild SA, Atkinson HJ, Urwin PE (2005) Cloning and characterisation of a Heterodera glycines aminopeptidase cDNA. Int J Parasitol 35: 1577-1585. doi:10.1016/j.ijpara.2005.07.017. PubMed: 16216247. 16216247

[39] Alkharouf NW, Klink VP, Matthews BF (2007) Identification of Heterodera glycines (soybean cyst nematode [SCN]) cDNA sequences with high identity to those of Caenorhabditis elegans having lethal mutant or RNAi phenotypes. Exp Parasitol 115: 247-258. doi:10.1016/j.exppara.2006.09.009. PubMed: 17052709. 17052709

[40] Bakhetia M, Urwin PE, Atkinson HJ (2007) QPCR analysis and RNAi define pharyngeal gland cell-expressed genes of Heterodera glycines required for initial interactions with the host. Mol Plant Microbe Interact 20: 306-312. doi:10.1094/MPMI-20-3-0306. PubMed: 17378433. 17378433

[41] Bakhetia M, Urwin PE, Atkinson HJ (2008) Characterisation by RNAi of pioneer genes expressed in the dorsal pharyngeal gland cell of Heterodera glycines and the effects of combinatorial RNAi. Int J Parasitol 38: 1589-1597. doi:10.1016/j.ijpara.2008.05.003. PubMed: 18579145. 18579145

[42] Dubreuil G, Magliano M, Deleury E, Abad P, Rosso MN (2007) Transcriptome analysis of root-knot nematode functions induced in the early stages of parasitism. New Phytol 176: 426-436. doi:10.1111/j.1469-8137.2007.02181.x. PubMed: 17692078. 17692078

[43] Adam MAM, Phillips MS, Jons JT, Blok VC (2008) Characterisation of the cellulose-binding protiens *Mj-cbp-1* of the root knot nematode, *Meloidogyne* *javanica* . Physiol Mol Plant Pathol 72: 21-28. doi:10.1016/j.pmpp.2008.05.002.

[44] Gleason CA, Liu QL, Williamson VM (2008) Silencing a candidate nematode effector gene corresponding to the tomato resistance gene Mi-1 leads to acquisition of virulence. Mol Plant Microbe Interact 21: 576-585. doi:10.1094/MPMI-21-5-0576. PubMed: 18393617. 18393617

[45] Tan JA, Jones MG, Fosu-Nyarko J (2013) Gene silencing in root lesion nematodes (Pratylenchus spp.) significantly reduces reproduction in a plant host. Exp Parasitol 133: 166-178. doi:10.1016/j.exppara.2012.11.011. PubMed: 23201220. 23201220

[46] Johnston MJ, McVeigh P, McMaster S, Fleming CC, Maule AG (2010) FMRFamide-like peptides in root knot nematodes and their potential role in nematode physiology. J Helminthol 84: 253-265. doi:10.1017/S0022149X09990630. PubMed: 19843350. 19843350

[47] Albertson DG, Thomson JN (1976) The pharynx of Caenorhabditis elegans. Philos Trans R Soc Lond B Biol Sci 275: 299-325. doi:10.1098/rstb.1976.0085. PubMed: 8805. 8805

[48] Rogers C, Reale V, Kim K, Chatwin H, Li C et al. (2003) Inhibition of Caenorhabditis elegans social feeding by FMRFamide-related peptide activation of NPR-1. Nat Neurosci 6: 1178-1185. doi:10.1038/nn1140. PubMed: 14555955. 14555955

[49] White JG, Southgate E, Thomson JN, Brenner S (1986) The structure of the nervous system of the nematode Caenorhabditis elegans. Philos Trans R Soc Lond B Biol Sci 314: 1-340. doi:10.1098/rstb.1986.0056. PubMed: 22462104. 22462104

[50] Cohen M, Reale V, Olofsson B, Knights A, Evans P et al. (2009) Coordinated regulation of foraging and metabolism in C. elegans by RFamide neuropeptide signaling. Cell Metab 9: 375-385. doi:10.1016/j.cmet.2009.02.003. PubMed: 19356718. 19356718

[51] Gray JM, Hill JJ, Bargmann CI (2005) A circuit for navigation in Caenorhabditis elegans. Proc Natl Acad Sci U S A 102: 3184-3191. doi:10.1073/pnas.0409009101. PubMed: 15689400. 15689400 PMC546636

[52] Tsalik EL, Hobert O (2003) Functional mapping of neurons that control locomotory behavior in Caenorhabditis elegans. J Neurobiol 56: 178-197. doi:10.1002/neu.10245. PubMed: 12838583. 12838583

[53] Wakabayashi T, Kitagawa I, Shingai R (2004) Neurons regulating the duration of forward locomotion in Caenorhabditis elegans. Neurosci Res 50: 103-111. doi:10.1016/j.neures.2004.06.005. PubMed: 15288503. 15288503

[54] Avery L, Horvitz HR (1989) Pharyngeal pumping continues after laser killing of the pharyngeal nervous system of C. elegans. Neuron 3: 473-485. doi:10.1016/0896-6273(89)90206-7. PubMed: 2642006. 2642006

[55] Avery L (1993) Motor neuron M3 controls pharyngeal muscle relaxation timing in Caenorhabditis elegans. J Exp Biol 175: 283-297. PubMed: 8440973. 8440973 10.1242/jeb.175.1.283

[56] Moffett CL, Beckett AM, Mousley A, Geary TG, Marks NJ et al. (2003) The ovijector of Ascaris suum: multiple response types revealed by Caenorhabditis elegans FMRFamide-related peptides. Int J Parasitol 33: 859-876. doi:10.1016/S0020-7519(03)00109-7. PubMed: 12865086. 12865086

