# Supplementary figures and images for: Utility of Host Delivered RNAi of Two FMRF Amide Like Peptides, flp-14 and flp-18, for the Management of Root Knot Nematode, Meloidogyne incognita
Source: PLoS One. 2013 Nov 6;8(11):e80603. doi: 10.1371/journal.pone.0080603 (PMC3819290; doi:10.1371/journal.pone.0080603)

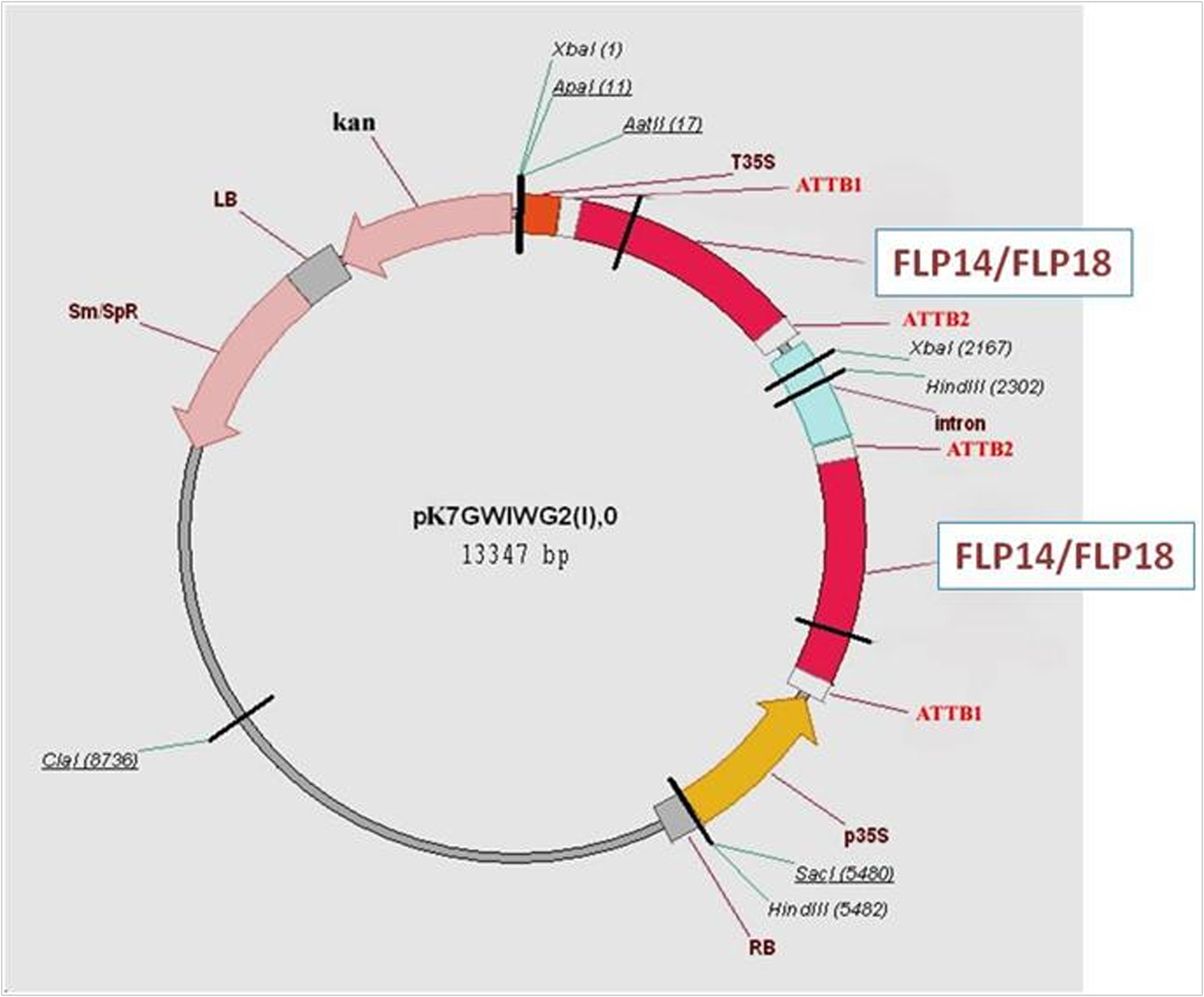

Supplement: Figure S1 — Hair pin RNAi constructs of flp-14 and flp-18 used for validation studies in tobacco. (TIF) [file pone.0080603.s001.tif]

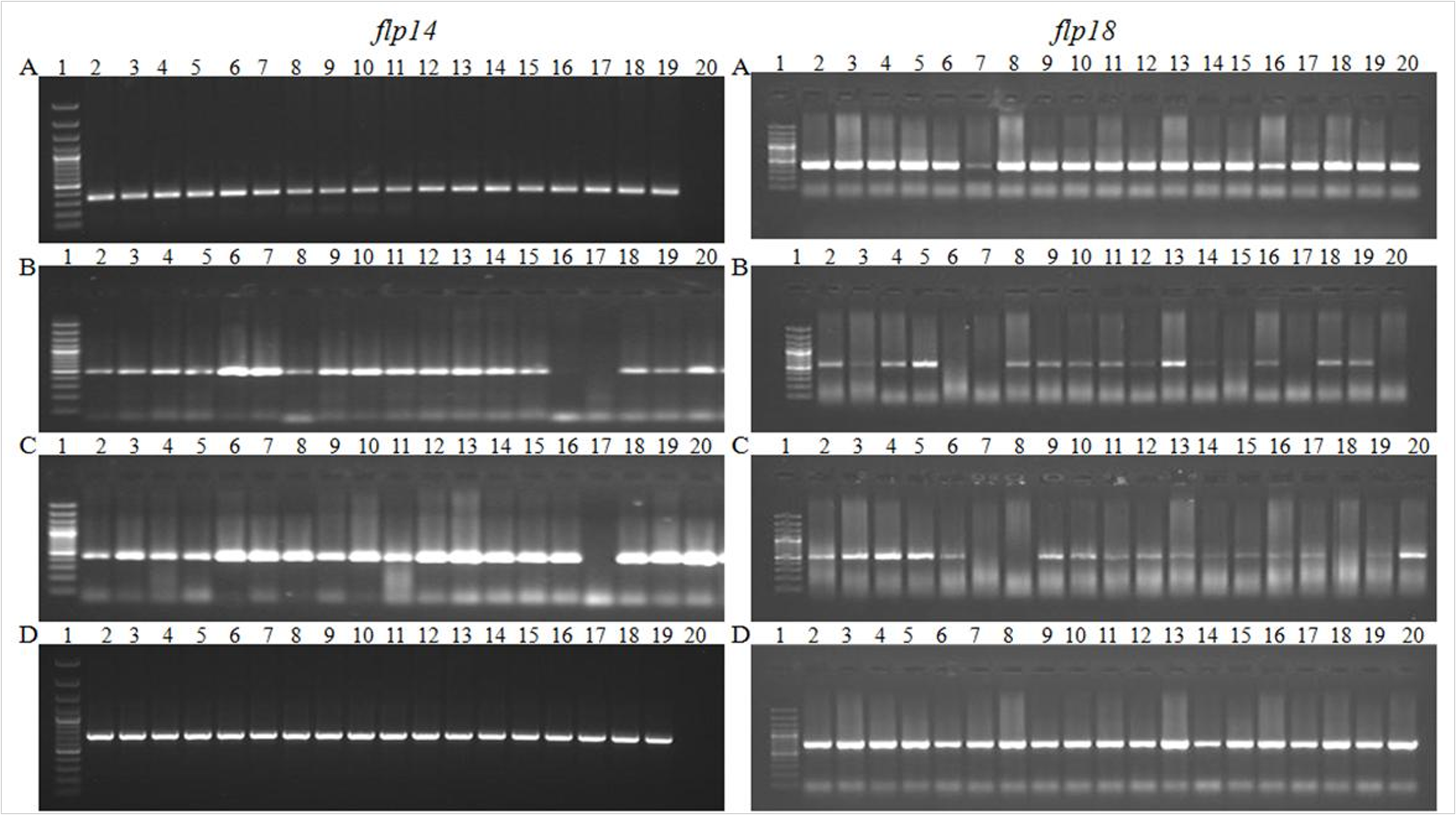

Supplement: Figure S3 — PCR analysis of T0 transgenic tobacco plants expressing dsRNA of flp-14 and flp-18. (A) Amplification of the target genes from T0 transgenic plants using gene specific primers. Lanes - 1: 100 bp DNA Ladder, 2-20: Primary events of flp-14 (342 bp), flp-18 (411 bp) (B) Amplification of sense strand in T0 transgenic events using primers to 35S promoter and attb2. Lanes - 1: 100 bp DNA Ladder, 2-20: Primary events of flp-14 (477 bp), flp-18 (546 bp). (C) Amplification of the antisense strand in T0 transgenic events using primers to amplify 35S terminator and attb2. Lanes - 1: 100 bp DNA Ladder, 2-20: Primary events of flp-14 (441 bp), flp-18 (510 bp). (D) Amplification of nptII gene from primary transgenic events. Lanes - 1: 100 bp DNA Ladder, 2-20: T0 events (750 bp). (TIF) [file pone.0080603.s003.tif]

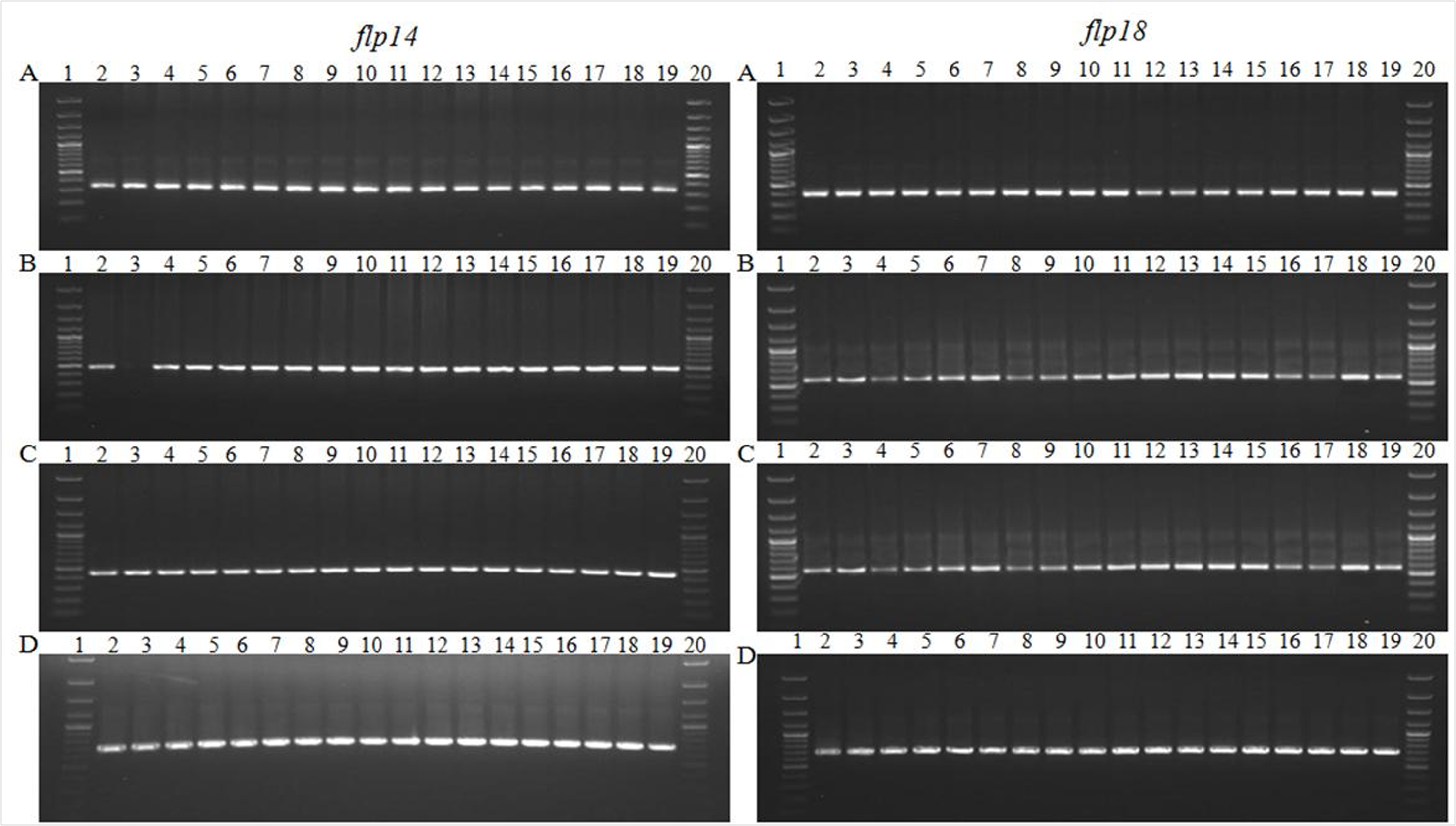

Supplement: Figure S4 — PCR analysis of T1 plants expressing dsRNA of flp-14 and flp-18. (A) Amplification of the target genes from T1 plants using gene specific primers. Lanes - 1: 100 bp DNA Ladder, 2-20: different T1 plants of flp-14 (342 bp), flp-18 (411 bp) (B) 35S promoter and the target gene fragment. Lanes - 1: 100 bp DNA Ladder, 2-20: different T1 events of flp-14 (477 bp), flp-18 (546 bp). (C) 35S terminator and target gene fragment from T1 transgenic events Lanes - 1: 100 bp DNA Ladder, 2-20: different T1 events of flp-14 (441 bp), flp-18 (510 bp). (D) nptII gene fragment. Lanes - 1: 100 bp DNA Ladder, 2-20: different T1 events (750 bp). (TIF) [file pone.0080603.s004.tif]
